# Supplementary material for: Non-fixation versus fixation of mesh in laparoscopic transabdominal preperitoneal repair of inguinal hernia: A systematic review and meta-analysis of randomized controlled trials
Source: PLoS One. 2024 Dec 6;19(12):e0314334. doi: 10.1371/journal.pone.0314334 (PMC11623461; doi:10.1371/journal.pone.0314334)
Supplement: S1 Appendix — (DOCX) [file pone.0314334.s002.docx]

**Appendix 1**

**Search strategies**

*Pubmed*

1. Hernia, Inguinal[Mesh]

2. Hernia, Femoral[Mesh]

3. inguinal hernia[Title/Abstract]

4. groin hernia[Title/Abstract]

5. 1 or 2 or 3 or 4

6. femoral hernia[Title/Abstract]

7. 5 or 6

8. laparoscopic repair[Title/Abstract]

9. laparoscopic hernia repair[Title/Abstract]

10. 8 or 9

11. transabdominal preperitoneal[Title/Abstract]

12. 10 or 11

13. TAPP[Title/Abstract]

14. 12 or 13

15. fixation[Title/Abstract]

16. 7 and 14 and 15

*Embase*

#1 'inguinal hernia'/exp

#2 'femoral hernia'/exp

#3 'inguinal hernia':ab,ti

#4 'groin hernia':ab,ti

#5 'femoral hernia':ab,ti

#6 #1 OR #2 OR #3 OR #4 OR #5

#7 'laparoscopic repair':ab,ti

#8 'laparoscopic hernia repair':ab,ti

#9 'transabdominal preperitoneal':ab,ti

#10 'tapp':ab,ti

#11 #7 OR #8 OR #9 OR #10

#12 'fixation':ab,ti

#13 #6 AND #11 AND #12

*CENTRAL*

#1 MeSH descriptor: [Hernia, Inguinal] explode all trees

#2 MeSH descriptor: [Hernia, Femoral] explode all trees

#3 (inguinal hernia):ti,ab,kw

#4 (groin hernia):ti,ab,kw

#5 (femoral hernia):ti,ab,kw

#6 #1 or #2 or #3 or #4 or #5

#7 (laparoscopic repair):ti,ab,kw

#8 (laparoscopic hernia repair):ti,ab,kw

#9 (transabdominal preperitoneal):ti,ab,kw

#10 (TAPP):ti,ab,kw

#11 (fixation):ti,ab,kw

#12 #7 or #8 or #9 or #10

#13 #6 and #12 and #11
