# Supplementary material for: Non-fixation versus fixation of mesh in laparoscopic transabdominal preperitoneal repair of inguinal hernia: A systematic review and meta-analysis of randomized controlled trials
Source: PLoS One. 2024 Dec 6;19(12):e0314334. doi: 10.1371/journal.pone.0314334 (PMC11623461; doi:10.1371/journal.pone.0314334)
Supplement: S1 File — (DOCX) [file pone.0314334.s003.docx]

| **number** | **Study** | **Reason for exclusion** |
| --- | --- | --- |
| 1 | Mesh fixation compared with nonfixation in transabdominal preperitoneal laparoscopic inguinal hernia repair. Surg Technol Int 23:122-125 | non-randomized trial |
| 2 | ARE THERE DIFFERENCES IN CHRONIC PAIN AFTER LAPAROSCOPIC INGUINAL HERNIA REPAIR USING THE TRANSABDOMINAL TECHNIQUE COMPARING WITH FIXATION OF THE MESH WITH STAPLES, WITH GLUE OR WITHOUT FIXATION? A CLINICAL RANDOMIZED, DOUBLE-BLIND TRIAL. Arq Bras Cir Dig 35:e1670 | **included** |
| 3 | Laparoscopic inguinal hernia repair using an anatomically contoured three-dimensional mesh. Surg Endosc 17(11):1784-1788 | non-randomized trial |
| 4 | Fixation vs non-fixation in laparoscopic hernia repair of inguinal hernia patients in St. Luke's Medical Center, Philippines: A cross-sectional study. Surgical Endoscopy and Other Interventional Techniques 31(2):S64 | conference Abstract |
| 5 | Mesh fixation in laparoscopic reconstruction of inguinal hernias. Rozhl Chir 98(7):282-286 | not in English |
| 6 | Changing the innate consensus about mesh fixation in trans-abdominal preperitoneal laparoscopic inguinal hernioplasty in adults: Short and long term outcome. Randomized controlled clinical trial. Int J Surg 83:117-124 | **included** |
| 7 | Comparison of mesh fixation and nonfixation in laparoscopic transabdominal preperitoneal repair of inguinal hernia. Formosan Journal of Surgery 52(6):212-220 | **included** |
| 8 | Long-term treatment results of inguinal hernias after using sutureless types of implantation. Russian Journal of Evidence-Based Gastroenterology 10(2):18-26 | not in English |
| 9 | Mesh fixation versus non fixation in laparoscopic transabdominal preperitoneal inguinal hernia repair. QJM 113(SUPPL 1):i98 | conference Abstract |
| 10 | The effect of transabdominal preperitoneal (TAPP) inguinal hernioplasty on chronic pain and quality of life of patients: mesh fixation versus non-fixation. Surg Endosc 31(10):4238-4243 | **included** |
| 11 | Our experience with tapp henioplasty without mesh fixation. Surgical Endoscopy and Other Interventional Techniques 28:S61 | conference Abstract |
| 12 | When is mesh fixation in TAPP-repair of primary inguinal hernia repair necessary? The register-based analysis of 11,230 cases. Surg Endosc 30(10):4363-4371 | non-randomized trial |
| 13 | Comparison of mesh fixation and non-fixation in transabdominal preperitoneal (TAPP) inguinal hernia repair: a randomized control trial. Surg Endosc. 2023;37: 5847-5854. | **included** |
| 14 | HIGHER REOPERATION RATES FOR TISSUE PENETRATING FIXATION METHODS IN LAPAROSCOPIC TRANSABDOMINAL PREPERITONEAL INGUINAL HERNIA REPAIR. British Journal of Surgery 109:vii14-vii15 | conference Abstract |
| 15 | Post-Operative Pain in Transabdominal Preperitoneal (TAPP) Hernia Repair: Mesh Fixation with Tacks Versus Non-Fixation. Pakistan Journal of Medical and Health Sciences 16(3):364-365 | full text not available |
| 16 | Laparoscopic transabdominal preperitoneal procedure with and without mesh-fixation for inguinal hernia repairs. International Journal of Clinical and Experimental Medicine 11(8):8651-8655 | full text not available |
| 17 | Effectiveness and safety of n-butyl-2-cyanoacrylate medical adhesive for noninvasive patch fixation in laparoscopic inguinal hernia repair. Surg Endosc 27(10):3792-3798 | non-randomized trial |
| 18 | Vacuum suction fixation versus staple fixation in TAPP laparoscopic hernia repair: introduction of a new technique for mesh fixation. Surg Endosc 30(1):114-120 | non-randomized trial |
| 19 | Stapled and nonstapled laparoscopic transabdominal preperitoneal (TAPP) inguinal hernia repair. A prospective randomized trial. Surg Endosc 13(8):804-806 | **Included** |
| 20 | International guidelines for groin hernia management. Hernia 22(1):1-165 | guideline |
| 21 | Transabdominal pre-peritoneal inguinal hernia repair with external fixation. Hernia 15(2):185-188 | No comparison |
| 22 | Patients satisfaction post laparoscopic transabdominal preperitoneal inguinal hernia repair with external fixation versus post internal fixation. Surgical Endoscopy and Other Interventional Techniques 29:S475 | Not about nonfixation |
| 23 | Laparoscopic hernia repair, where are we? Surgical Endoscopy and Other Interventional Techniques 25:S73 | review |
| 24 | Comparison between mesh fixation and non-fixation in patients undergoing total extraperitoneal inguinal hernia repair. Niger J Clin Pract 23(7):897-899 | TEP |
| 25 | Glue vs. Tacks in Mesh Fixation for Minimally Invasive Inguinal Hernia Repair â€“ A Randomised Controlled Trial. http://trialsearchwhoint/Trial2aspx?TrialID=ACTRN12620000742976 | Trial registry record |
| 26 | Experience of laparoscopic total extraperitoneal inguinal hernia repair without fixation of the mesh. Pakistan Journal of Medical and Health Sciences 4(4) | TEP |
| 27 | [Pain and quality of life after laparoscopic transabdominal preperitoneal hernioplasty with different ways of fixing the peritoneum. Results of early completion randomized clinical trial]. Khirurgiia (Mosk)(9):14-20 | Not about fixation of mesh |
| 28 | Dulucq's technique for laparoscopic totally extraperitoneal hernioplasty. Journal of Minimal Access Surgery 16(1):94-96 | TEP |
| 29 | Lightweight partially absorbable monofilament mesh (polypropylene/ poliglecaprone 25) for TAPP inguinal hernia repair: Initial experience. Surgical Laparoscopy, Endoscopy and Percutaneous Techniques 17(2):91-94 | No comparison |
| 30 | Transabdominal laparoscopic inguinal hernia repair: Is there a place for biological mesh? Hernia 12(6):609-612 | Not about fixation of mesh |
| 31 | Laparoscopic transabdominal inguinal hernia repair: A randomized study of fibrin sealant versus absorbable tack to fix the mesh. Surgical Endoscopy and Other Interventional Techniques 29:S35 | Not about nonfixation of mesh |
| 32 | Post-operative pain and early complications in patients undergoing laparoscopic transabdominal preperitoneal inguinal hernia repair using mesh fixation with sutures versus tackers. Pakistan Journal of Medical and Health Sciences 15(1):537-539 | Not about nonfixation of mesh |
| 33 | Laparoscopic transabdominal repair of incarcerated recurrent inguinal hernia. Surgical Endoscopy and Other Interventional Techniques 31(2):S462 | No comparison |
| 34 | Fixation free laparoscopic obliteration of inguinal hernia defects with the 3D dynamic responsive scaffold ProFlor. Sci Rep 12(1):18971 | No comparison |
| 35 | Physiologic Cyclical Load on Inguinal Hernia Scaffold ProFlor Turns Biological Response into Tissue Regeneration. Biology (Basel) 12(3) | Not about fixation of mesh |
| 36 | First-in-man permanent laparoscopic fixation free obliteration of inguinal hernia defect with the 3D dynamic responsive implant ProFlor-E®. Case report. International Journal of Surgery Case Reports 77:S2-S7 | Not about fixation of mesh |
| 37 | Mesh fixation methods and chronic pain after transabdominal preperitoneal (TAPP) inguinal hernia surgery: a comparison between fibrin sealant and tacks. Surgical Endoscopy 31(10):4077-4084 | Not about nonfixation of mesh |
| 38 | Meta-analysis of randomized trials comparing tissue adhesives versus mechanical mesh fixation in laparoscopic inguinal hernia repair. Surgical endoscopy and other interventional techniques 30:S8 | review |
| 39 | Meta-analysis of randomized trials comparing tissue adhesives versus mechanical mesh fixation in laparoscopic inguinal hernia repair. Surgical Endoscopy and Other Interventional Techniques 30:S8 | review |
| 40 | Meta-analysis of randomized trials comparing nonpenetrating vs mechanical mesh fixation in laparoscopic inguinal hernia repair. American Journal of Surgery 211(1):239-249 | review |
| 41 | Groin hernia repair by laparoscopic techniques: current status and controversies. World J Surg 29(8):1052-1057 | review |
| 42 | TAPP repair of inguinal hernia: Learning curve and complications in a general hospital. Pakistan Journal of Medical and Health Sciences 12(3):1002-1004 | Not about fixation of mesh |
| 43 | Laparoscopic hernia repairs without mesh fixation. Hernia 22(1):S181 | No comparison |
| 44 | Comparison of Early Outcomes in Patients Undergoing Suture Fixation Versus Tack Fixation of Mesh in Laparoscopic Transabdominal Preperitoneal (TAPP) Repair of Inguinal Hernia. Cureus 14(7):e26821 | Not about nonfixation of mesh |
| 45 | Laparoscopic inguinal hernia repair: over a thousand convincing reasons to go on. Hernia 12(5):493-498 | review |
| 46 | An experience of over 1500 cases of laparoscopic groin hernia surgery at a tertiary care centre. Surgical Endoscopy 33:S370 | Not about fixation of mesh |
| 47 | Single large 30 × 15 polipropilene mesh in tapp laparoscopic bilateral inguinal repair. An option for medial bilateral recurrence. Hernia 22(1):S191 | Not about fixation of mesh |
| 48 | Methods of mesh fixation in open and laparoscopic approach in inguinal hernia repair. Restrospective study in 195 patients. Revista Hispanoamericana de Hernia 8(1):3-10 | No comparison of mesh |
| 49 | Early outcomes of self-adhering vs. conventional polyester mesh in laparoscopic inguinal hernia repair. Surgical Endoscopy and Other Interventional Techniques 30:S405 | Not about nonfixation of mesh |
| 50 | Laparoscopic inguinal hernia repair using an anatomically contoured three-dimensional mesh. Surgical Endoscopy 17(11):1784-1788 | Not about nonfixation of mesh |
| 51 | Post-operative pain after TAPP: Impact of mesh fixation methods. Hernia 20:S200 | review |
| 52 | Dysejaculation after laparoscopic inguinal herniorrhaphy: a nationwide questionnaire study. Surg Endosc 26(4):979-983 | No comparison |
| 53 | TAPP: Recurrence prevention: 5 crucial procedural steps. Hernia 21(2):S154 | Technical details |
| 54 | Lightweight mesh and noninvasive fixation: an effective concept for prevention of chronic pain with laparoscopic hernia repair (TAPP). Surg Endosc 24(12):2958-2964 | No comparison |
| 55 | Technique of laparoscopic hernioplasty (TAPP). Chirurgische Gastroenterologie Interdisziplinar 16(2):98-103 | Technical details |
| 56 | One-year results of a prospective, randomised clinical trial comparing four meshes in laparoscopic inguinal hernia repair (TAPP). Hernia 15(5):503-510 | Not about fixation of mesh |
| 57 | Early postoperative and one year results of a randomized controlled trial comparing the impact of extralight titanized polypropylene mesh and traditional heavyweight polypropylene mesh on pain and seroma production in laparoscopic hernia repair (TAPP). World J Surg 35(8):1791-1797 | Not about fixation of mesh |
| 58 | Intravesical migration of a polypropylene mesh 3 years after laparoscopic transperitoneal hernioplasty. Urologe - Ausgabe A 41(4):366-368 | Case report |
| 59 | [Intravesical migration of a polypropylene mesh implant 3 years after laparoscopic transperitoneal hernioplasty]. Urologe A 41(4):366-368 | Case report |
| 60 | Pain after laparascopic bilateral hernioplasty : Early results of a prospective randomized double-blind study comparing fibrin versus staples. Surg Endosc 22(5):1206-1209 | Not about nonfixation of mesh |
| 61 | Development of a two port laparoscopic inguinal hernia repair without need for Tacks using a vertical approach for peritoneal flap. European Surgery - Acta Chirurgica Austriaca 48(1):S107 | Not about fixation of mesh |
| 62 | Transabdominal Preperitoneal (TAPP) inguinal hernia repair using a vertical peritoneal flap. Surgical Endoscopy and Other Interventional Techniques 31:S146 | Not about fixation of mesh |
| 63 | Laparoscopic transabdominal preperitoneal self-adhesive mesh repair for laterally placed ventral and incisional hernias. Surgical Endoscopy and Other Interventional Techniques 30:S63 | Not about fixation of mesh |
| 64 | Does self-fixing mesh extend indications to transabdominal pre-peritoneal laparoscopic hernia repair (TAPP)? Surgical Endoscopy and Other Interventional Techniques 30:S63 | Not about nonfixation of mesh |
| 65 | Laparoscopic Inguinal Hernia Repair With a Novel Hernia Mesh Incorporating a Nitinol Alloy Frame Compared With a Standard Lightweight Polypropylene Mesh. Surgical innovation 22(5):508‐513 | Not about nonfixation of mesh |
| 66 | Femoral vs inguinal hernia repair: Long-term clinical and quality of life (QOL) outcomes from a prospective, international database. Surgical Endoscopy and Other Interventional Techniques 28:355 | Not about nonfixation of mesh |
| 67 | Objective hypoesthesia and pain after transabdominal preperitoneal hernioplasty: a prospective, randomized study comparing tissue adhesive versus spiral tacks. Surgical endoscopy and other interventional techniques 26(4):1079‐1085 | Not about nonfixation of mesh |
| 68 | Objective hypoesthesia and pain after transabdominal preperitoneal hernioplasty: a prospective, randomized study comparing tissue adhesive versus spiral tacks. Surg Endosc 26(4):1079-1085 | Not about nonfixation of mesh |
| 69 | Feasibility and safety in bilateral transumbilical laparoscopic inguinal hernia repair. Surgical Endoscopy and Other Interventional Techniques 25:S5 | Not about nonfixation of mesh |
| 70 | Transumbilical three port laparoscopic inguinal hernia repair - Does it justify reduced costs? Surgical Endoscopy and Other Interventional Techniques 25:S6 | Not about nonfixation of mesh |
| 71 | Reduced port laparoscopic tep repair for groin hernia: An effective approach. Surgical Endoscopy and Other Interventional Techniques 26:S55 | TEP |
| 72 | [Comparison between two different mesh fixation methods in laparoscopic inguinal hernia repair: tacker vs. Synthetic cyanoacrylate glue.]. Minerva Chir 69(6):321-329 | Not about nonfixation of mesh |
| 73 | Comparison of mesh fixation and non-fixation in laparoscopic totally extraperitoneal inguinal hernia repair. Hernia 21(4):543‐548 | TEP |
| 74 | Tapp laparoscopic repair for inguinal hernia using glue fixation - Our initial experience. Surgical Endoscopy and Other Interventional Techniques 31(2):S318 | Not about nonfixation of mesh |
| 75 | Comparison of self-gripping mesh with mesh fixation with fibrin-glue in laparoscopic hernia repair (TAPP). Bratisl Lek Listy 113(2):103-107 | Not about nonfixation of mesh |
| 76 | Prospective randomized study comparing single-incision versus multi-trocar laparoscopic totally extraperitoneal inguinal hernia repair at 2 years. Surgical endoscopy 32:S439 | TEP |
| 77 | Prospective randomized study comparing single-incision laparoscopic versus multi-trocar laparoscopic totally extraperitoneal (TEP) inguinal hernia repair at 2 years. Surgical endoscopy 32(7):3262‐3272 | TEP |
| 78 | Mesh Migration and Bowel Perforation as a Late Complication of Transabdominal Preperitoneal Laparoscopic Hernia Repair. Cureus 14(12):e32683 | Case report |
| 79 | A modified access technique combining TAPP and TEP to facilitate extraperitoneal inguinal hernia repair using MINI-instruments. Surgical Endoscopy and Other Interventional Techniques 26:S326 | Not about nonfixation of mesh |
| 80 | Minilaparoscopic technique for inguinal hernia repair combining transabdominal pre-peritoneal and totally extraperitoneal approaches. Jsls 16(4):569-575 | Not about nonfixation of mesh |
| 81 | Comparing fibrin sealant with staples for mesh fixation in laparoscopic transabdominal hernia repair: a case control-study. Surg Endosc 22(3):668-673 | Not about nonfixation of mesh |
| 82 | Transfascial suture fixation technique in laparoscopic repair of inguinal hernia. Asian J Endosc Surg 13(2):246-249 | Not about nonfixation of mesh |
| 83 | Comparison of the Clinical Outcome and Complications in Laparoscopic Hernia Repair of Inguinal Hernia With Mesh Fixation Using Fibrin Glue vs Tacker. Indian J Surg 78(6):464-470 | Not about nonfixation of mesh |
| 84 | Outcomes of laparoscopic totally extraperitoneal inguinal hernia repair (lap TEP) with fixation device (ProTack™) and self fixating mesh (ProGrip™). Surgical Endoscopy 33:S370 | TEP |
| 85 | PATIENT REPORTED OUTCOMES FOLLOWING LAPAROSCOPIC GROIN HERNIA REPAIR IN A DISTRICT GENERAL HOSPITAL USING TWO DIFFERENT METHODS OF MESH FIXATION. British Journal of Surgery 109:vii7-vii8 | Not about nonfixation of mesh |
| 86 | The influence in chronic post-herniotomy pain and quality of life with xation versus no xation of mesh in TAPP hernia repair. http://trialsearchwhoint/Trial2aspx?TrialID=ChiCTR-INR-16009427 | Trial registry record |
| 87 | A Randomized Controlled Clinical Trial comparing Self-gripping Mesh with Medical Adhesive Mesh in Laparoscopic Transabdominal Preperitoneal Hernioplasty. http://trialsearchwhoint/Trial2aspx?TrialID=ChiCTR1800017360 | Trial registry record |
| 88 | A Randomized Controlled Clinical Trial comparing Medical Adhesive Mesh Fixation with Non-fixation in Laparoscopic Transabdominal Preperitoneal Hernioplasty. http://trialsearchwhoint/Trial2aspx?TrialID=ChiCTR1800014307 | Trial registry record |
| 89 | A novel technique using mesh to repair a recurrent large indirect inguinoscrotal hernia. World Journal of Laparoscopic Surgery 14(1):65-67 | Not about nonfixation of mesh |
| 90 | Recurrent hernia following endoscopic total extraperitoneal repair. J Laparoendosc Adv Surg Tech A 13(1):21-25 | Not about nonfixation of mesh |
| 91 | Less pain after tack fixation of mesh than with self-gripping mesh following laparoscopic inguinal hernia repair: a randomized clinical trial. British journal of surgery 108(SUPPL 8):viii5 | Not about nonfixation of mesh |
| 92 | Update on fixation techniques and outcome in laparoscopic inguinal hernia (TAPP): south American experience. Hernia 20(1):S41 | No comparison |
| 93 | Prospective, randomized and controlled study of mesh displacement after laparoscopic inguinal repair: fixation versus no fixation of mesh. Surgical endoscopy 30(3):1134‐1140 | TEP |
| 94 | Laparoscopic management of iatrogenic bladder injury and bladder stone formation following laparoscopic inguinal herniorrhaphy. Hernia 12(4):429-430 | No comparison |
| 95 | Comparing non-fixation of mesh to mesh fixation in laparoscopic inguinal hernia repair. http://trialsearchwhoint/Trial2aspx?TrialID=CTRI/2009/091/000020 | Trial registry record |
| 96 | To compare and see which is better, two ways of fixing the mesh in laparoscopic inguinal hernia mesh repair. http://trialsearchwhoint/Trial2aspx?TrialID=CTRI/2019/11/022154 | Trial registry record |
| 97 | A Comparison between laparoscopic repair of inguinal hernia by using two different mesh. http://trialsearchwhoint/Trial2aspx?TrialID=CTRI/2019/05/019006 | Trial registry record |
| 98 | Comparison of pain after Laparoscopic repair of hernia when Self Gripping mesh is used as opposed to mesh fixed with absorbable tackers when performing the surgery. http://trialsearchwhoint/Trial2aspx?TrialID=CTRI/2022/10/046213 | Trial registry record |
| 99 | Comparison of the clinical outcomes of self-gripping mesh versus staple fixation mesh in laparoscopic inguinal hernia repair. Ann Ital Chir 94:82-89 | Not about nonfixation of mesh |
| 100 | Laparoscopic Techniques in Treatment of Inguinal Hernia in Patients with Peritoneal Dialysis: Experiences from 15 Cases. J Laparoendosc Adv Surg Tech A | Not about nonfixation of mesh |
| 101 | A novel glue device for fixation of mesh and peritoneal closure during laparoscopic inguinal hernia repair: short- and medium-term results. European Surgery - Acta Chirurgica Austriaca 49(1):27-31 | Not about nonfixation of mesh |
| 102 | COLE-TAPP-LIRA. IS IT POSSIBLE? HOW TO DO IT! Surgical Endoscopy 36(2):S622 | Not about nonfixation of mesh |
| 103 | An analysis of results in a single-blinded, prospective randomized controlled trial comparing non-fixating versus self-fixating mesh for laparoscopic inguinal hernia repair. Surgical endoscopy 33(8):2670‐2679 | TEP |
| 104 | Endoscopic skills for groin hernia repair can be learned and trained in the lab: Presentation of a new TAPP-teacher. Hernia 21(2):S201 | Not about nonfixation of mesh |
| 105 | Robotic inguinal hernia repair (rTAPP): Anatomy lessons and results of a casuistic of 302 operated hernias. British Journal of Surgery 108(SUPPL 8):viii48 | Not about nonfixation of mesh |
| 106 | Laparoscopic inguinal hernia repair via totally extraperitoneal approach (TEP), under general or epidural anesthesia. Surgical Endoscopy 33(2):S588 | TEP |
| 107 | Comparative study between the use of self-fixating mesh and non self-fixating mesh in laprascopic inguinal hernia repair transabdominal preperitoneal (TAPP) technique. QJM 113(SUPPL 1):i51 | Not about nonfixation of mesh |
| 108 | Fibrin glue fixation of bioactive extracellular matrix mesh compared with soft prolene mesh for laparoscopic hernia repair. Surg Laparosc Endosc Percutan Tech 18(6):569-572 | Not about nonfixation of mesh |
| 109 | Robotic Inguinal Hernia Repair. Surg Technol Int 36:99-104 | Not about nonfixation of mesh |
| 110 | Robotic Inguinal Hernia Repair - Update 2022 during the SARS-COVID-19 Pandemic. Surg Technol Int 42 | Not about nonfixation of mesh |
| 111 | Experience of using n-butyl cyanoacrylate mesh fixation in laparoscopic groin hernia repair: Influence on the recurrence. Hernia 21(2):S233 | Not about nonfixation of mesh |
| 112 | Combined TAPP and TEP: A new modified technique for laparoscopic inguinal hernia repair. World Journal of Laparoscopic Surgery 5(2):72-75 | Not about nonfixation of mesh |
| 113 | Factors guarantee competence of laparoscopic repair of inguinal hernia. World Journal of Laparoscopic Surgery 11(3):124-127 | Not about nonfixation of mesh |
| 114 | Meta-analysis of laparoscopic groin hernia repair with or without mesh fixation. International Journal of Surgery 71:190-199 | review |
| 115 | Meta-analysis of laparoscopic groin hernia repair with or without mesh fixation. Int J Surg 71:190-199 | review |
| 116 | Meta-analysis of randomized trials comparing laparoscopic repair of groin hernia with or without mesh fixation. British Journal of Surgery 106:31 | review |
| 117 | MRI visible meshes and non-invasive fixation: Case serie. Hernia 21(2):S240 | Not about nonfixation of mesh |
| 118 | Relaparoscopic treatment of recurrences after previous laparoscopic inguinal hernia repair. Minim Invasive Surg 2013:260131 | Not about nonfixation of mesh |
| 119 | Preperitoneal closed-system suction drainage after totally extraperitoneal hernioplasty in the prevention of early seroma formation: a prospective double-blind randomised controlled trial. Hernia 22(3):455‐465 | Not about nonfixation of mesh |
| 120 | [Differentiated approach to preperitoneal alloplasty for complex recurrent inguinal hernias]. Klin Khir(3):17-20 | Not about nonfixation of mesh |
| 121 | Evaluation of TAPP efficacy and combined fixation of mesh implants for inguinal hernia repairs. Hernia 21(2):S233 | Not about nonfixation of mesh |
| 122 | TAPP with combined ultrapro mesh fixation at inguinal hernias. Hernia 20:S202 | Not about nonfixation of mesh |
| 123 | A calcified foreign body in the bladder due to an unusual complication after laparoscopic incisional hernia repair. Surg Laparosc Endosc Percutan Tech 21(1):e28-30 | Case report |
| 124 | Causes of recurrence after laparoscopic hernioplasty. A multicenter study. Surg Endosc 12(3):226-231 | Not about nonfixation of mesh |
| 125 | Feasibility of incremental laparoscopic inguinal hernia repair development in China: an 11-year experience. J Am Coll Surg 216(2):258-265 | Not about nonfixation of mesh |
| 126 | Fibrin sealant for mesh fixation in laparoscopic groin hernia repair does not increase long-term recurrence. Surg Endosc 30(3):986-992 | Not about nonfixation of mesh |
| 127 | Self-gripping mesh versus fibrin glue fixation in laparoscopic inguinal hernia repair: a randomized prospective clinical trial in young and elderly patients. Open Med (Wars) 11(1):497-508 | Not about nonfixation of mesh |
| 128 | Fibrin glue versus stapler fixation in laparoscopic transabdominal inguinal hernia repair: a single center 5-year experience and analysis of the results in the elderly. Int J Surg 12 Suppl 2:S94-s98 | Not about nonfixation of mesh |
| 129 | Simulated training model in a low cost for laparoscopic inguinal hernioplasty. Acta Cirurgica Brasileira 36(1):1-7 | Not about nonfixation of mesh |
| 130 | Laparoscopic herniorrhaphy. Surgical Clinics of North America 72(5):1109-1124 | Not about nonfixation of mesh |
| 131 | Laparoscopic herniorrhaphy. Surg Clin North Am 72(5):1109-1124 | Not about nonfixation of mesh |
| 132 | Small bowel obstruction owing to displaced spiral tack after laparoscopic TAPP inguinal hernia repair. Surg Laparosc Endosc Percutan Tech 20(3):e132-135 | Case report |
| 133 | One surgeon's experience with r-TAPP: a retrospective analysis of 150 consecutive robotic inguinal hernia cases. J Robot Surg 16(5):1151-1155 | No comparison |
| 134 | Comparison of a New Self-Gripping Mesh with Other Fixation Methods for Laparoscopic Hernia Repair in a Rat Model. Journal of the American College of Surgeons 209(4):543-544 | Animal experiment |
| 135 | Use of human fibrin glue (tissucol) versus staples for mesh fixation in laparoscopic transabdominal preperitoneal hernioplasty [1]. Annals of Surgery 246(5):903 | Not about nonfixation of mesh |
| 136 | Spray application of fibrin sealant with an angled spray tip device in laparoscopic inguinal hernia repair. European Surgery - Acta Chirurgica Austriaca 42(4):171-176 | Not about nonfixation of mesh |
| 137 | The impact of atraumatic fibrin sealant vs. staple mesh fixation in TAPP hernia repair on chronic pain and quality of life: results of a randomized controlled study. Surgical endoscopy 26(1):249‐254 | Not about nonfixation of mesh |
| 138 | HerniaSurge guidelines: Mesh fixation in open and laparo-endoscopic inguinal/femoral hernia repair. Hernia 20:S172-S173 | guidelines |
| 139 | Self-gripping mesh versus staple fixation in laparoscopic inguinal hernia repair: a prospective comparison. Surg Endosc 27(5):1798-1802 | Not about nonfixation of mesh |
| 140 | [Outcomes of various techniques of mesh prosthesis fixation in laparoscopic hernia repair]. Khirurgiia (Mosk)(1):34-41 | Not about nonfixation of mesh |
| 141 | Evaluation of cause of recurrence after laparoscopic groin hernia surgery: TAPP. Hernia 21(2):S234 | Not about nonfixation of mesh |
| 142 | Impact of mesh type on recurrence in robotic-assisted inguinal hernia repair. Hernia 21(1):S95 | Not about nonfixation of mesh |
| 143 | Ambulant intraperitoneal onlay mesh repair of inguinal hernias using dual mesh plus fixed by helical stapler: 14 years results. Surgical Endoscopy and Other Interventional Techniques 28:S61 | Not about nonfixation of mesh |
| 144 | Mesh fixation compared to non-fixation in total extraperitoneal inguinal hernia repair: a randomized controlled trial in a rural hospital setting. Surgical endoscopy and other interventional techniques 25:S215 | TEP |
| 145 | Mesh fixation compared to nonfixation in total extraperitoneal inguinal hernia repair: a randomized controlled trial in a rural center in India. Surgical endoscopy 25(10):3300‐3306 | TEP |
| 146 | [Ultrasonic geometry of synthetic endoprostheses after transabdominal preperitoneal hernioplasty of inguinal hernias]. Khirurgiia (Mosk)(1):53-60 | Not about nonfixation of mesh |
| 147 | Ultrasound Appearance of Mesh After Transabdominal Preperitoneal Inguinal Hernia Repair. J Laparoendosc Adv Surg Tech A 30(4):395-401 | Not about fixation of mesh |
| 148 | TAPP inguinal hernia repair with parietex ProGrip™ self-fixating mesh. Hernia 20:S207 | No comparison |
| 149 | IS THERE A CORRELATION BETWEEN TYPE OF PROSTHESIS FIXATION IN T.A.P.P. AND AFTER SURGERY RECOVERY? OUR CENTER EXPERIENCE. British Journal of Surgery 109:vii10-vii11 | Not about nonfixation of mesh |
| 150 | Endoscopic extraperitoneal inguinal hernia repair with double mesh: Indications, technique, complications, and results. Journal of Laparoendoscopic and Advanced Surgical Techniques - Part A 15(6):586-590 | Not about fixation of mesh |
| 151 | Endoscopic extraperitoneal inguinal hernia repair with double mesh: indications, technique, complications, and results. J Laparoendosc Adv Surg Tech A 15(6):586-590 | Not about fixation of mesh |
| 152 | Technique of suturing the mesh in laparoscopic total extra peritoneal (TEP) repair of inguinal hernia. Surgeon 2(5):264-272 | TEP |
| 153 | Recurrence after groin hernia repair-revisited. Int J Surg 11(5):374-377 | No comparison |
| 154 | Initial experience in laparoscopic bilateral inguinal hernia repair (TEP) with new anatomical mesh with large pore and low weight (Dynamesh Endolap) in short stay (6 months follow-up). Ambulatory Surgery 22(3):89-91 | TEP |
| 155 | Initial experience in laparoscopic bilateral inguinal hernia repair (Tep) with new anatomical mesh with large pore and row weight (dynamesh endolap) in short stay (6 months follow-up). Ambulatory Surgery 21(2):93 | TEP |
| 156 | [Using surgical mesh of a new construction in laparoscopic treatment of inguinal hernias. Comparative study]. Klin Khir(7):42-45 | Not about fixation of mesh |
| 157 | New meshes with nitinol frame in laparoscopic repair of recurrent inguinal hernia. Surgical endoscopy and other interventional techniques 26:S7 | Not about fixation of mesh |
| 158 | Endoscopic Hernia Repair: A Novel Technique for the Repair of Inguinal Hernia in a Cadaver Model. Surg Laparosc Endosc Percutan Tech 31(4):404-407 | Not about fixation of mesh |
| 159 | Mesh fixation techniques for laparoscopic inguinal hernia repair in adults. Cochrane Database of Systematic Reviews 2017(10) | review |
| 160 | Optiview ''555 manish technique'' for TEP repair: An innovative technique. Surgical Endoscopy 33:S463-S465 | TEP |
| 161 | Changing the innate consensus about mesh fixation in trans-abdominal preperitoneal laparoscopic inguinal hernioplasty in adults: short and long term outcome. Randomized controlled clinical trial. International journal of surgery (London, England) 83:117‐124 | Not about nonfixation of mesh |
| 162 | Changing the innate consensus about mesh fixation in trans-abdominal preperitoneal laparoscopic inguinal hernioplasty in adults: Short and long term outcome. Randomized controlled clinical trial. International Journal of Surgery 83:117-124 | Not about nonfixation of mesh |
| 163 | Totally extraperitoneal laparoscopic hernioplasty: The optimal surgical approach. Surgical Laparoscopy, Endoscopy and Percutaneous Techniques 19(6):501-505 | TEP |
| 164 | Small bowel lesion due to spiral tacks after laparoscopic intraperitoneal onlay mesh repair for incisional hernia. Int J Surg Case Rep 4(3):283-285 | incisional hernia |
| 165 | Mesh erosion into the urinary bladder following laparoscopic inguinal hernia repair; is this the tip of the iceberg? Hernia 14(3):317-319 | Not about fixation of mesh |
| 166 | Assessment of feasibility and safety of cyanoacrylate glue versus absorbable tacks for inguinal hernia mesh fixation. A prospective comparative study. Wideochir Inne Tech Maloinwazyjne 18(1):90-98 | Not about nonfixation of mesh |
| 167 | Minimally Invasive Approaches to Inguinal Hernias. Surg Clin North Am 98(3):637-649 | review |
| 168 | Comparison of post-operative pain after laparoscopic total extraperitoneal mesh repair of indirect inguinal hernia with tacker. Pakistan journal of medical and health sciences 15(10):2733‐2735 | TEP |
| 169 | Thou shalt not trust online videos for inguinal hernia repair techniques. Surg Endosc 35(10):5724-5728 | Not about nonfixation of mesh |
| 170 | Laparoscopic Transabdominal Preperitoneal (TAPP) Inguinal Hernia Repair Using Fibrin Glue for Fixation of the Mesh and Peritoneum Closure. Surg Laparosc Endosc Percutan Tech 30(4):e24-e27 | Not about nonfixation of mesh |
| 171 | Laparoscopic inguinal hernia repair: cost-effectiveness analysis of trend modifications of the technique. Updates Surg 73(5):1945-1953 | Not about nonfixation of mesh |
| 172 | Laparoscopic Transabdominal Preperitoneal (TAPP) Inguinal Hernia Repair Using Fibrin Glue for Fixation of the Mesh and Peritoneum Closure. Surgical Laparoscopy, Endoscopy and Percutaneous Techniques 30(4):E24-E27 | Not about nonfixation of mesh |
| 173 | comparison of post operative outcomes after two methods of mesh fixation in laparoscopic inguinal hernia repair. http://trialsearchwhoint/Trial2aspx?TrialID=IRCT201202118972N1 | Trial registry record |
| 174 | Comparison of different method at mesh fixation technique Transabdominal per-peritoneal(TAPP) laparoscopy surgery in inguinal hernia. http://trialsearchwhoint/Trial2aspx?TrialID=IRCT20100620004219N1 | Trial registry record |
| 175 | Fixation or non-fixation of mesh in laparoscopic inguinal hernia repair. http://trialsearchwhoint/Trial2aspx?TrialID=IRCT20190204042618N4 | Trial registry record |
| 176 | Comparison of Inguinal Hernia Repair with Laparoscopic and open Methods. http://trialsearchwhoint/Trial2aspx?TrialID=IRCT20200727048228N1 | Trial registry record |
| 177 | Comparison of the effect of fix and non-fix mesh (Use of external fixation (hernia band) after surgery) in causing complications after hernioplasty surgery. http://trialsearchwhoint/Trial2aspx?TrialID=IRCT20200825048515N25 | Trial registry record |
| 178 | Comparison of mesh fixation and non-fixation in laparoscopic inguinal hernia repair. http://trialsearchwhoint/Trial2aspx?TrialID=IRCT20210224050491N1 | Trial registry record |
| 179 | Comparison of mesh fixation and non-fixation in laparoscopic Transabdominal prepritoneal inguinal hernia repair. http://trialsearchwhoint/Trial2aspx?TrialID=IRCT20210309050650N2 | Trial registry record |
| 180 | The Impact of Fibrin fixation of macroporous mesheS in TransAbdominal PrePeritoneal hernia repair. http://trialsearchwhoint/Trial2aspx?TrialID=ISRCTN41994541 | Trial registry record |
| 181 | Study comparing single-incision laparoscopy versus multitrocar laparoscopy totally extraperitoneal inguinal hernia repair (TEP) at 2 years. http://trialsearchwhoint/Trial2aspx?TrialID=ISRCTN63754528 | Trial registry record |
| 182 | Cyanoacrylate Glue Versus Absorbable Tacks in Mesh Fixation for Laparoscopic Extraperitoneal Inguinal Hernia Repair: a Randomized Controlled Trial. Surgical laparoscopy, endoscopy & percutaneous techniques 31(3):291‐297 | TEP |
| 183 | Effective inguinal ring closure methods to prevent recurrence after laparoscopic inguinal hernia repair for indirect inguinal hernias with large inguinal rings. Surgical Endoscopy 33:S374 | Not about nonfixation of mesh |
| 184 | Transabdominal preperitoneal (TAPP) inguinal hernia repair using meshes without fixation. Eesti Arst 94:28 | No comparison |
| 185 | New activities to the IAAS basket. Ambulatory Surgery 23(2):50-51 | review |
| 186 | Randomised controlled trial of n-butyl cyanoacrylate glue fixation versus suture fixation of mesh in laparoscopic totally extraperitoneal hernia repair. Journal of minimal access surgery 12(2):118‐123 | Not about nonfixation of mesh |
| 187 | Laparoscopic repair of inguinal hernias. Preliminary results in 162 cases. Annales de Chirurgie 48(6):507-511 | No comparison |
| 188 | Preliminary results of a single-blinded, prospective randomized controlled trial compairing non-fixating vs. self-fixating mesh for laparoscopic inguinal hernia repair. Surgical endoscopy and other interventional techniques 31:S127 | TEP |
| 189 | An analysis of results in a single-blinded, prospective randomized controlled trial comparing non-fixating vs. self-fixating mesh for laparoscopic inguinal hernia repair. Surgical endoscopy and other interventional techniques 32(1):S2 | TEP |
| 190 | Initial experience with the use of N-butyl 2-cyanoacrylate glue for the fixation of polypropylene mesh in laparoscopic hernia repair. Surg Laparosc Endosc 8(4):291-293 | No comparison |
| 191 | Prevention and management of intraoperative complication during single incision laparoscopic totally extraperitoneal repair. J Minim Invasive Surg 25(1):36-39 | TEP |
| 192 | A new technique for laparoscopic hernia repair using fibrin sealant. Surg Technol Int 12:120-126 | Not about nonfixation of mesh |
| 193 | Use of fibrin sealant for prosthetic mesh fixation in laparoscopic extraperitoneal inguinal hernia repair. Ann Surg 233(1):18-25 | Not about nonfixation of mesh |
| 194 | [Our experience with totally extraperitoneal technique of laparoscopic inguinal hernia repairs modified by Stolzenburg]. Magy Seb 67(3):129-134 | TEP |
| 195 | The role of fibrin glue in decreasing chronic pain in laparoscopic totally extraperitoneal (TEP) inguinal hernia repair: a single surgeon's experience. ANZ J Surg 81(3):154-158 | TEP |
| 196 | Transumbilical single-incision laparoscopic inguinal hernia repair - Feasibility study on anatomical specimens. Wideochirurgia I Inne Techniki Maloinwazyjne 5(2):72-75 | Not about fixation of mesh |
| 197 | Mesh fixation in TAPP laparoscopic hernia repair: introduction of a new method in a prospective randomized trial. Surg Endosc 28(2):531-536 | Not about nonfixation of mesh |
| 198 | Self-fixating mesh in tapp technique. Possibility of reduction of the chronic pain and recurrence rate. Results of a prospective, single centre study. Surgical Endoscopy and Other Interventional Techniques 31(2):S441 | Not about nonfixation of mesh |
| 199 | Innovation in Laparoscopic Inguinal Hernia Reparation - Initial Experiences with the Parietex Progrip Laparoscopic(™) - Mesh. Front Surg 2:28 | Not about nonfixation of mesh |
| 200 | Usage of a self-adhesive mesh in TAPP hernia repair: A prospective study based on Herniamed Register. J Minim Access Surg 12(3):226-234 | Not about fixation of mesh |
| 201 | Usage of a self adhesive mesh in TAPP hernia repair. A prospective study based on Herniamed® register. Hernia 20(1):S50 | Not about fixation of mesh |
| 202 | Reduction of chronic post-herniotomy pain and recurrence rate. Use of the anatomical self-gripping ProGrip laparoscopic mesh in TAPP hernia repair. Preliminary results of a prospective study. Wideochir Inne Tech Maloinwazyjne 10(3):373-381 | Not about fixation of mesh |
| 203 | Use of an anatomical self-gripping Progrip™ laparoscopic mesh in TAPP hernia repair. Possible standard? Preliminary results of a prospective study. European Surgery - Acta Chirurgica Austriaca 48:149-154 | Not about fixation of mesh |
| 204 | Randomized prospective study of totally extraperitoneal inguinal hernia repair: fixation versus no fixation of mesh. JSLS : Journal of the Society of Laparoendoscopic Surgeons 10(4):457‐460 | TEP |
| 205 | TEP. Hernia 21(2):S153-S154 | TEP |
| 206 | TEP for elective primary unilateral inguinal hernia repair in men: what do we know? Hernia 23(3):439-459 | TEP |
| 207 | Seroma following transabdominal preperitoneal patch plasty (TAPP): incidence, risk factors, and preventive measures. Surg Endosc 32(5):2222-2231 | Not about fixation of mesh |
| 208 | Early Surgical Intervention following Inguinal Hernia Repair with Severe Postoperative Pain. Front Surg 4:67 | Not about fixation of mesh |
| 209 | Robotic tapp hernia and sports hernia repair based on twenty five-year experience of laparoscopic tep. Surgical Endoscopy and Other Interventional Techniques 31(2):S465 | Not about fixation of mesh |
| 210 | Laparoscopic preperitoneal mesh repair using a novel self-adhesive mesh. Journal of Minimal Access Surgery 7(3):192-194 | Not about fixation of mesh |
| 211 | Laparoscopic preperitoneal mesh repair using a novel self-adhesive mesh. J Minim Access Surg 7(3):192-194 | Not about fixation of mesh |
| 212 | Laparoscopic repair of large inguinoscrotal hernias with combined use of plug and flat mesh. Hippokratia 25(1):38-41 | Not about fixation of mesh |
| 213 | COMPARATIVE EVALUATION OF TAPP HERNIOPLASTY WITH USE OF VARIOUS METHODS OF FIXING THE RETICULAR ENDOPROSTHESIS AND TEP IN THE TREATMENT OF INGUINAL HERNIAS. Georgian Med News(278):15-20 | Not about nonfixation of mesh |
| 214 | TAPP modification for large and gigantic inguinal hernia repair. European Surgery - Acta Chirurgica Austriaca 47:S222 | Not about nonfixation of mesh |
| 215 | Causes of recurrence in laparoscopic inguinal hernia repair. J Minim Access Surg 2(3):187-191 | Not about nonfixation of mesh |
| 216 | Efficiency and safety of mesh fixation in laparoscopic inguinal hernia repair using n-butyl cyanoacrylate: long-term biocompatibility in over 1,300 mesh fixations. Hernia 16(2):153-162 | Not about nonfixation of mesh |
| 217 | Comparative analysis of the tapp and tep in the treatment of inguinal hernias: 1 year observation period. Surgical Endoscopy and Other Interventional Techniques 31(2):S62 | Not about nonfixation of mesh |
| 218 | Comparative analysis of the TAPP and TEP in the treatment of inguinal hernias: 1 year observation period. Hernia 21(2):S215 | Not about nonfixation of mesh |
| 219 | Non-fixation Versus Fixation of Mesh in Totally Extraperitoneal Repair of Inguinal Hernia: a Comparative Study. Indian J Surg 80(2):128-133 | TEP |
| 220 | Initial experience with the use of fibrin sealant for the fixation of the prosthetic mesh in laparoscopic transabdominal preperitoneal hernia repair. Rozhl Chir 84(8):399-402 | Not about nonfixation of mesh |
| 221 | Post operative pain in laparoscopic groin hernia repair using a self-gripping mesh versus stapler fixation: a randomized double blinded study. Surgical endoscopy 32:S437 | Not about nonfixation of mesh |
| 222 | Efficacy of Laparoscopic Iliopubic Tract Repair Plus Transabdominal Preperitoneal Hernioplasty for Treating Inguinal Hernia After Robot-assisted Radical Prostatectomy. Surg Laparosc Endosc Percutan Tech 33(3):276-281 | Not about fixation of mesh |
| 223 | Are postoperative complaints and complications influenced by different techniques in fashioning and fixing the mesh in transperitoneal laparoscopic hernioplasty? Results of a prospective randomized trial. World journal of surgery 26(12):1481‐1484 | Not about nonfixation of mesh |
| 224 | Laparoscopic resorbable mesh fixation. Assessment of an innovative disposable instrument delivering resorbable fixation devices: I-Clip™. Final results of a prospective multicentre clinical trial. Hernia 12(2):177-183 | Not about nonfixation of mesh |
| 225 | Laparoscopic resorbable mesh fixation. Assessment of an innovative disposable instrument delivering resorbable fixation devices: I-Clip(TM). Final results of a prospective multicentre clinical trial. Hernia 12(2):177-183 | Not about nonfixation of mesh |
| 226 | Minimally invasive approaches to inguinal hernia repair. J Long Term Eff Med Implants 20(2):105-116 | Not about nonfixation of mesh |
| 227 | The Management of Indirect Inguinal Hernia Sac in Laparoscopic Inguinal Hernia Repair: A Systemic Review of Literature. Surg Laparosc Endosc Percutan Tech 31(5):645-653 | review |
| 228 | Intraoperative adjunctive techniques to reduce seroma formation in laparoscopic inguinal hernioplasty: a systematic review. Hernia 23(4):723-731 | review |
| 229 | How I do it: the horizontal-bilateral unfolding method for self-gripping (Progrip™) mesh placement in laparoscopic inguinal hernia repair. Hernia 23(4):809-815 | Not about nonfixation of mesh |
| 230 | Perineal hernia repair after abdominoperineal resection (APR) with the laparoscopic-peritoneal dual fixation technique. Hernia 26(5):1307-1314 | Not about nonfixation of mesh |
| 231 | 3D meshfixationby using medical glue in TAPP. European Surgery - Acta Chirurgica Austriaca 47:S208 | Not about nonfixation of mesh |
| 232 | The effect of transabdominal preperitoneal (TAPP) inguinal hernioplasty on chronic pain and quality of life of patients: mesh fixation versus non-fixation. Surgical endoscopy and other interventional techniques:1‐6 | No relevant data |
| 233 | Tackers versus glue mesh fixation: an objective assessment of postoperative acute and chronic pain using inflammatory markers. Hernia 21(4):549‐554 | Not about nonfixation of mesh |
| 234 | Can a Fully Articulating Electromechanical Laparoscopic Needle Driver Compare with a Robotic Platform in Transabdominal Preperitoneal Inguinal Hernia Repair? J Laparoendosc Adv Surg Tech A 32(11):1164-1169 | Not about nonfixation of mesh |
| 235 | [Supravesical hernia as a rare cause of inguinal herniation and its laparoscopic treatment with TAPP approach]. Rozhl Chir 95(8):322-324 | Not about nonfixation of mesh |
| 236 | Does the use of monopolar energy as the preferred mode of dissection effectively reduce seroma formation in laparoscopic total extra peritoneal hernioplasty? A prospective double-blinded randomized control trial. Hernia 24(4):821‐829 | TEP |
| 237 | [Analysis of recurrent factors and therapeutic effect after laparoscopic inguinal hernia repair]. Zhonghua Wai Ke Za Zhi 61(6):507-510 | Not about nonfixation of mesh |
| 238 | Single port endo-laparoscopic surgery (SPES) for multiple procedures. Surgical Endoscopy and Other Interventional Techniques 24(1):S677 | Not about nonfixation of mesh |
| 239 | An in-vitro comparison of three different fixation methods for tapp technique: Non-inferiority trial. Hernia 21(1):S73 | Not about nonfixation of mesh |
| 240 | Use of human fibrin glue (Tissucol) versus staples for mesh fixation in laparoscopic transabdominal preperitoneal hernioplasty: a prospective, randomized study. Ann Surg 245(2):222-231 | Not about nonfixation of mesh |
| 241 | Application of Straight-needle, Three-tailed, Knot-free, Peritoneal Sutures in Laparoscopic Transabdominal Preperitoneal Hernia Repair. J Vis Exp(177) | Not about nonfixation of mesh |
| 242 | Tapp hernioplasty without mesh fixation. Surgical Endoscopy and Other Interventional Techniques 31(2):S317 | No comparison |
| 243 | SHORT-TERM RESULTS OF LAPAROSCOPIC TOTALLY EXTRAPERITONEAL (TEP) GROIN HERNIA REPAIR WITH LIGHTWEIGHT FLAT MESH WITHOUT FIXATION. AGAINST THE ESTABLISHED? British Journal of Surgery 110:ii45 | TEP |
| 244 | Laparoscopic Inguinal Hernia Repair Without General Anesthesia. Surgical Endoscopy and Other Interventional Techniques 30:S246 | Not about nonfixation of mesh |
| 245 | The current management of patients with inguinal hernia in Spain and Latin America compared to the International Guide for the Management of Inguinal Hernia. Revista Hispanoamericana de Hernia 10(2):70-81 | review |
| 246 | Is mesh fixation necessary during laparoscopic transabdominal preperitoneal (TAPP) hernia repair? A surgeon's 6 year experience and literature review. Surgical Endoscopy and Other Interventional Techniques 29:S307-S308 | review |
| 247 | A randomized clinical trial comparing early patient-reported pain after open anterior mesh repair versus totally extraperitoneal repair of inguinal hernia. Br J Surg 108(12):1433-1437 | TEP |
| 248 | Robot assisted management of mesh erosion into the urinary bladder following bilateral inguinal hernia repair. BJU International 131:44-45 | Not about fixation of mesh |
| 249 | Simulated training model in a low cost for laparoscopic inguinal hernioplasty. Acta Cir Bras 36(1):e360108 | Not about fixation of mesh |
| 250 | Laparoscopic hernia repair--complications. Jsls 2(1):35-40 | Not about nonfixation of mesh |
| 251 | Total extra-peritoneal repair of groin hernia: prospective evaluation at a tertiary care center. Hernia 12(1):65-71 | TEP |
| 252 | TAPP surgery with mesh fixation and peritoneal closure using n-butyl-2-cyanoacrylate (LiquiBand®FIX8TM)—initial experience. European Surgery - Acta Chirurgica Austriaca 48(2):110-114 | Not about nonfixation of mesh |
| 253 | TAPP groin hernia repair with 3D mesh fixed with histoacrylic glue: Early and long term results of 55 patients. Hernia 20:S197-S198 | Not about nonfixation of mesh |
| 254 | The change in groin pain perception after transabdominal preperitoneal inguinal hernia repair with glue fixation: a prospective trial of a single surgeon's experience. Surg Endosc 32(10):4284-4289 | Not about nonfixation of mesh |
| 255 | Assessment of Intra-Operative Difficulties and Early Post-Operative Complications in Laparoscopic Inguinal Hernia Repair using Conventional Polypropylene Mesh Versus 3D Mesh. QJM : monthly journal of the Association of Physicians 114(SUPPL 1) | Not about nonfixation of mesh |
| 256 | Management and advantages of new self-adhesive mesh in laparoscopic inguinal hernia repair. Surgical Endoscopy and Other Interventional Techniques 30:S160 | Not about fixation of mesh |
| 257 | New prosthetic material and fixing method: For laparoscopic repair of large hiatal hernias. Surgical Endoscopy and Other Interventional Techniques 29:S296 | hiatal hernia |
| 258 | Laparoscopic preperitoneal inguinal hernia repair using preformed polyester mesh without fixation: prospective study with 1-year follow-up results in a rural setting. Surg Laparosc Endosc Percutan Tech 18(1):33-39 | No comparison |
| 259 | Laparoscopic preperitoneal inguinal hernia repair using preformed polyester mesh without fixation: Prospective study with 1-year follow-up results in a rural setting. Surgical Laparoscopy, Endoscopy and Percutaneous Techniques 18(1):33-39 | No comparison |
| 260 | Use of human fibrin glue (Tisseel) versus staples for mesh fixation in laparoscopic transabdominal preperitoneal hernioplasty (TISTA): a randomized controlled trial (NCT01641718). BMC Surg 14:18 | Not about nonfixation of mesh |
| 261 | A new technique to avoid unintentional adhesion while deploying ProGrip mesh and its utility in the laparoscopic repair of obturator hernia. Journal of Minimal Access Surgery 17(1):116-119 | No comparison |
| 262 | A prospective, multicenter, observational study on quality of life after laparoscopic inguinal hernia repair with ProGrip laparoscopic, self-fixating mesh according to the European Registry for Abdominal Wall Hernias Quality of Life Instrument. Surgery (united states) 160(5):1344‐1357 | No comparison |
| 263 | Prospective study on QOL after laparoscopic inguinal hernia repair with progrip™ Laparoscopic self-fixating mesh with the EuraHS-QOL instrument. Surgical endoscopy and other interventional techniques 31(2):S312 | No comparison |
| 264 | Feasibility of a novel tacking method of securing mesh in transabdominal preperitoneal inguinal hernia repair: Secure tacking against recurrence. Asian J Endosc Surg 11(4):385-391 | Not about nonfixation of mesh |
| 265 | Mesh fixation with takers or no fixation in laparoscopic transabdominal preperitoneal (TAPP) inguinal hernia repair. A prospective trial. Surgical Endoscopy and Other Interventional Techniques 29:S464 | Conference Abstract |
| 266 | Laparoscopic inguinal hernia repair: a rare case of colonic mesh migration. Ann R Coll Surg Engl 100(8):e1-e4 | Case report |
| 267 | Mesh shrinkage is the potential pathogenesis of chronic somatic pain following transabdominal preperitoneal repair: Report of two cases. Asian J Endosc Surg 14(4):798-802 | Case report |
| 268 | Emergent Robotic Incarcerated Large Scrotal Inguinal Hernia Repair. Surgical Endoscopy 36(1 SUPPL):S77 | Robotic surgery |
| 269 | Laparoscopic Ventral Hernia Repair With Elastomeric Pain Pump. http://clinicaltrialsgov/show/NCT00472134 | Trial registry record |
| 270 | Stapler Versus Glue for Laparoscopic Groin Hernia Repair. http://clinicaltrialsgov/show/NCT00793286 | Trial registry record |
| 271 | AbsorbaTack Evaluation of Postoperative Pain Following Laparoscopic Hernia Repair. http://clinicaltrialsgov/show/NCT00749268 | Trial registry record |
| 272 | Functional Outcome After Incisional Hernia Repair: open Versus Laparoscopic Repair. http://clinicaltrialsgov/show/NCT00625053 | Trial registry record |
| 273 | Fibrin Glue Versus Tacked Fixation in Groin Hernia Repair (TAPP). http://clinicaltrialsgov/show/NCT01000116 | Trial registry record |
| 274 | Comparing Non-fixation of Mesh to Mesh Fixation in Laparoscopic Inguinal Hernia Repair. http://clinicaltrialsgov/show/NCT01117337 | Trial registry record |
| 275 | Use of Human Fibrin Glue Versus Staples for Mesh Fixation in Laparoscopic Transabdominal Preperitoneal Hernioplasty. http://clinicaltrialsgov/show/NCT01641718 | Trial registry record |
| 276 | Fibrin Sealant Spray Versus Mechanical Stapling in Laparoscopic Totally Extraperitoneal Hernioplasty. http://clinicaltrialsgov/show/NCT01727050 | Trial registry record |
| 277 | TEP Versus Open Minimal Suture Repair for the Sportsman's Groin. http://clinicaltrialsgov/show/NCT02297711 | Not about fixation of mesh |
| 278 | Comparison of Self-Fixating vs Non-Fixating Hernia Mesh. http://clinicaltrialsgov/show/NCT02062775 | Trial registry record |
| 279 | Comparing Pain After Laparoscopic Hernia Repair Using Two Different Types of Mesh Fixation. http://clinicaltrialsgov/show/NCT02467140 | Trial registry record |
| 280 | Study of Laparoscopic Inguinal Hernia Repair. http://clinicaltrialsgov/show/NCT02920307 | Trial registry record |
| 281 | Lichtenstein Versus TAPP and TEP in Groin Hernioplasty. http://clinicaltrialsgov/show/NCT02928146 | Trial registry record |
| 282 | Comparison of Different Meshes in Laparoscopic Hernia Repair. http://clinicaltrialsgov/show/NCT02712827 | Trial registry record |
| 283 | Mesh Position and Outcomes Following Inguinal Hernia Repair Using an MRI Visible Hernia Mesh. http://clinicaltrialsgov/show/NCT02770703 | Trial registry record |
| 284 | Laparoscopic Groin Hernia Repair by a 3D ENDOLAP Visible Mesh With or Without LiquiBand Fix 8 Mesh Fixation. http://clinicaltrialsgov/show/NCT02781870 | Trial registry record |
| 285 | The Role of the Robotic Platform in Inguinal Hernia Repair Surgery. http://clinicaltrialsgov/show/NCT02816658 | Trial registry record |
| 286 | Comparison of Self Fixating Mesh to Mesh Fixation With Metallic Tacks in Laparoscopic Inguinal Hernia Repair. http://clinicaltrialsgov/show/NCT03564535 | Trial registry record |
| 287 | Conventional Versus Robot Assisted Laparoscopic Inguinal Hernia Repair. http://clinicaltrialsgov/show/NCT03904888 | Trial registry record |
| 288 | A Clinical Study to Evaluate the Clinical Performance and Safety of LiquiBand FIX8® Versus AbsorbaTack™ for Hernia Mesh Fixation and Peritoneal Closure in Groin Hernia Repair. http://clinicaltrialsgov/show/NCT04009213 | Trial registry record |
| 289 | Short Term Outcome of Laparoscopic Trans-abdominal Preperitoneal Inguinal Hernia Repair Without Mesh Fixation. http://clinicaltrialsgov/show/NCT04532983 | Trial registry record |
| 290 | Different Methods of Mesh Fixation (Mechanical Versus Non Mechanical) in TAPP Inguinal Hernioplasty in Adults. http://clinicaltrialsgov/show/NCT04272424 | Trial registry record |
| 291 | Laparscopic Inguinal Hernia Repair - Does the Choice of Self-fixated Mesh Matter? http://clinicaltrialsgov/show/NCT05091853 | Trial registry record |
| 292 | Is it Necessary to Fix Mesh in Laparoscopic Hernia Repair. http://clinicaltrialsgov/show/NCT05152654 | Trial registry record |
| 293 | Laparoscopic Transabdominal Preperitoneal Surgical Repair of Inguinal Hernia Using Sutured Repair Versus Tacker Use. http://clinicaltrialsgov/show/NCT05574751 | Trial registry record |
| 294 | A Randomized Controlled Trial Studying the Fibrin Glue vs Metallic Tackers in Mesh Fixation During TAPP Repair. http://clinicaltrialsgov/show/NCT05466084 | Trial registry record |
| 295 | Outcomes of Mesh Fixation Versus Non Fixation in Laparoscopic TAPP Inguinal Hernia Repair. http://clinicaltrialsgov/show/NCT05430984 | Trial registry record |
| 296 | Inversion and Fixation of the Transversalis Fascia in Laparoscopic Inguinal Hernia Repair. http://clinicaltrialsgov/show/NCT05420818 | Trial registry record |
| 297 | MESH MIGRATION IN TOTAL EXTRAPERITONEAL INGUINAL HERNI REPAIR. http://clinicaltrialsgov/show/NCT05655988 | Trial registry record |
| 298 | Post-operative Pain Control-TAP Block Using Exparel vs. Marcaine for Hernia Repairs. http://clinicaltrialsgov/show/NCT05177991 | Trial registry record |
| 299 | MEsh FIxation in Laparoendoscopic Repair of Large M3 Inguinal Hernias. http://clinicaltrialsgov/show/NCT05678465 | Trial registry record |
| 300 | Initial experience using a handheld fully articulating software-driven laparoscopic needle driver in TAPP inguinal hernia repair. Surg Endosc 35(6):3221-3231 | Not about fixation of mesh |
| 301 | What are the influencing factors for chronic pain following TAPP inguinal hernia repair: an analysis of 20,004 patients from the Herniamed Registry. Surg Endosc 32(4):1971-1983 | Not about fixation of mesh |
| 302 | Robotic-Assisted Transabdominal Preperitoneal Ventral Hernia Repair. Surg Technol Int 36:95-97 | Robotic surgery |
| 303 | Mesh fixation with fibrin glue versus tacker in laparoscopic totally extraperitoneal inguinal hernia repair. ANZ J Surg 91(10):2086-2090 | TEP |
| 304 | Association of different mesh and mesh fixation combinations with reoperation risk after laparoscopic groin hernia surgery. a swedish hernia registry study of 25,190 tep and tapp repairs. British Journal of Surgery 108(SUPPL 8):viii11 | No comparison |
| 305 | Association of Mesh and Fixation Options with Reoperation Risk after Laparoscopic Groin Hernia Surgery: A Swedish Hernia Registry Study of 25,190 Totally Extraperitoneal and Transabdominal Preperitoneal Repairs. J Am Coll Surg 234(3):311-325 | No comparison |
| 306 | Comparison of peritoneal closure techniques in laparoscopic transabdominal preperitoneal inguinal hernia repair: a prospective randomized study. Hernia 19(6):879-885 | Not about fixation of mesh |
| 307 | Experimental comparison of type of Tissucol dilution and composite mesh (Parietex) for laparoscopic repair of groin and abdominal hernia: observational study conducted in a university laboratory. Hernia 11(3):211-215 | Not about fixation of mesh |
| 308 | Fibrin glue for mesh fixation in laparoscopic transabdominal preperitoneal (TAPP) hernia repair: indications, technique, and outcomes. Surg Endosc 20(12):1846-1850 | Not about nonfixation of mesh |
| 309 | [Use of fibrin glue (Tissucol) for mesh fixation in laparoscopic transabdominal hernia repair]. Chir Ital 57(6):753-759 | Not about nonfixation of mesh |
| 310 | Laparoscopic repair of inguinal hernias using an intraperitoneal onlay mesh technique and a Parietex composite mesh fixed with fibrin glue (Tissucol). Personal technique and preliminary results. Surg Endosc 21(11):1961-1964 | Not about nonfixation of mesh |
| 311 | Quantification of pain in laparoscopic transabdominal preperitoneal (TAPP) inguinal hernioplasty identifies marked differences between prosthesis fixation systems. Surgery 142(1):40-46 | Not about nonfixation of mesh |
| 312 | Surgical technique and outcome of hybrid tapp for complicated inguinal hernia. Surgical Endoscopy 33(2):S720 | No comparison |
| 313 | The transversus abdominis plane block may reduce chronic postoperative pain one year after TAPP ingunial hernia repair. Ann Med Surg (Lond) 55:190-194 | Not about fixation of mesh |
| 314 | Transabdominal pre-peritoneal inguinal hernioplasty (TAPP) without mesh fixation. Initial experience in the short term. Revista Hispanoamericana de Hernia 8(1):19-24 | No comparison |
| 315 | Cost comparison of fibrin sealant versus tack screws for mesh fixation in laparoscopic repair of inguinal hernia. Hosp Pract (1995) 46(4):233-237 | Not about nonfixation of mesh |
| 316 | Prospective evaluation of the economic benefit of laparoscopic transabdominal preperitoneal (TAPP) hernia repair in the detection of occult bilateral inguinal hernias. Surgical Endoscopy and Other Interventional Techniques 26:S327 | Not about fixation of mesh |
| 317 | Effect of direct defect closure on post-operative seroma during tep inguinal hernia repair. Surgical endoscopy 32:S489 | TEP |
| 318 | TAPP after recurrent TEP. Surgical Endoscopy and Other Interventional Techniques 30:S69 | Not about fixation of mesh |
| 319 | Comparison between self-gripping, semi re-absorbable meshes with polyethylene meshes in lichtenstein, tensionfree hernia repair: preliminary results from a single center. Acta bio-medica 89(1):72‐78 | Lichtenstein surgery |
| 320 | The impact of atraumatic fibrin sealant vs. staple mesh fixation in tapp hernia repair on chronic pain and quality of life - Results of a randomized controlled study. Surgical Endoscopy and Other Interventional Techniques 24(1):S618 | Not about nonfixation of mesh |
| 321 | Use of fibrin glue for fixation of mesh and approximation of Peritoneum in Transabdominal Preperitoneal (TAPP) inguinal hernia repair: technical feasibility and early surgical outcomes. Surg Laparosc Endosc Percutan Tech 24(2):e43-45 | Not about nonfixation of mesh |
| 322 | Use of fibrin glue for fixation of mesh and approximation of peritoneum in transabdominal preperitoneal (TAPP) inguinal hernia repair: Technical feasibility and early surgical outcomes. Surgical Laparoscopy, Endoscopy and Percutaneous Techniques 24(2):e43-e45 | Not about nonfixation of mesh |
| 323 | Mid-Term Follow Up of TAPP Hernia Repair Without Staples and Glue: An Audit of the Data. Surg Technol Int 26:151-154 | No comparison |
| 324 | Tapp Groin Hernia Repair with progrip™ and V-loc™ 180. The Fastest Way and Without Pain. European Surgical Research 49(3-4):197-198 | Not about nonfixation of mesh |
| 325 | Incarcerated femoral hernia in women-A critical view on approach options. European Surgery - Acta Chirurgica Austriaca 51(1):S40 | Not about fixation of mesh |
| 326 | Suture and Fixation of the Transversalis Fascia during Robotic-Assisted Transabdominal Preperitoneal Hernia Repair to Prevent Seroma Formation after Direct Inguinal Hernia Repair. Surg Innov 28(3):284-289 | Robotic-Assisted TAPP |
| 327 | Cost-effective of laparoscopic versus open groin hernia surgery in Colombia: An institutional perspective. Journal of Surgical Research 172(2):289 | Not about fixation of mesh |
| 328 | Fast Early Recovery After Transabdominal Preperitoneal Repair in Athletes with Sportsman's Groin: A Prospective Clinical Cohort Study. J Laparoendosc Adv Surg Tech A 27(3):272-276 | Not about fixation of mesh |
| 329 | [Positioning of mesh implants in endoscopic inguinal hernia repair]. Khirurgiia (Mosk)(6):53-59 | No comparison |
| 330 | No difference in sexual dysfunction after transabdominal preperitoneal (TAPP) approach for inguinal hernia with fibrin sealant or tacks for mesh fixation. Surg Endosc 31(2):661-666 | Not about nonfixation of mesh |
| 331 | The efficacy of absorbable versus non-absorbable fixation in laparoscopic totally extraperitoneal (tep) repair of large inguinal hernias. Asian J Surg 42(12):995-1000 | TEP |
| 332 | Robotic hernia repair: Indian experience. Hernia 21(2):S158-S159 | Robotic hernia repair |
| 333 | Laparoscopic inguinal hernia repair: Prospective comparison between standard and self-gripping mesh. Surgical Endoscopy and Other Interventional Techniques 27:S163 | Not about fixation of mesh |
| 334 | Laparoscopic inguinal hernia repair: Optimal technical variations and results in 1700 cases. American Surgeon 66(9):848-852 | Not about fixation of mesh |
| 335 | Laparoscopic inguinal hernia repair: optimal technical variations and results in 1700 cases. Am Surg 66(9):848-852 | Not about fixation of mesh |
| 336 | A comparative follow up study of transabdominal preperitoneal mesh repair in inguinal hernias with or without mesh fixation. J Pak Med Assoc 71(1(a)):28-30 | Not RCT |
| 337 | Laparoscopic management of mesh migration into urinary bladder following laparoscopic totally extraperitoneal inguinal hernia repair—A case report. International Journal of Surgery Case Reports 78:401-404 | case report |
| 338 | Laparoscopic management of mesh migration into urinary bladder following laparoscopic totally extraperitoneal inguinal hernia repair-A case report. Int J Surg Case Rep 78:401-404 | case report |
| 339 | One surgeon, one centre: 3 years of experience for glue mesh fixation in TAPP hernia repair. Hernia 20:S216 | Not about nonfixation of mesh |
| 340 | Laparoscopic approach for the treatment of chronic groin pain after inguinal hernia repair. Surgical Endoscopy and Other Interventional Techniques 31:S41 | Not about fixation of mesh |
| 341 | Transabdominal Preperitoneal (TAPP) for the Treatment of Spigelian hernias. Jsls 25(2) | Not about nonfixation of mesh |
| 342 | Transabdominal preperitoneal approach to incisional border hernia repair. Hernia 21(2):S190 | Not about fixation of mesh |
| 343 | Fixation in inguinal open and endoscopic repair. Hernia 21(2):S147 | review |
| 344 | Current status of laparoscopic inguinal hernia repair in Denmark. Hernia 12(6):583-587 | review |
| 345 | Danish Hernia Database recommendations for the management of inguinal and femoral hernia in adults. Dan Med Bull 58(2):C4243 | review |
| 346 | A prospective randomized trial comparing the rebound hernia repair device and lightweight mesh for laparoscopic inguinal hernia: An interim analysis. Surgical Endoscopy and Other Interventional Techniques 25:S325 | Not about fixation of mesh |
| 347 | Intracorporeal suturing of mesh and peritoneum during TAPP as substituting for Tackers. QJM 113(SUPPL 1):i88 | Not about nonfixation of mesh |
| 348 | Sufficient fastening of mesh for inguinal hernia repair: One tack on the pubic tubercle: 110 patient meta-analysis. Surgical Endoscopy and Other Interventional Techniques 29:S327 | Not about nonfixation of mesh |
| 349 | Preliminary results of robotic inguinal hernia repair following its introduction in a single-center trial. Ann Gastroenterol Surg 4(4):441-447 | robotic inguinal hernia repair |
| 350 | Complications Related to Laparoscopic Technique Fixation of Mesh among Inguinal Hernia Patients. NeuroQuantology 20(10):4296-4303 | Not about nonfixation of mesh |
| 351 | Robotic Inguinal Hernia Repair After Prostatectomy: How to Navigate Safely. Surg Laparosc Endosc Percutan Tech 32(1):66-72 | Robotic Inguinal Hernia Repair |
| 352 | Robotic Inguinal Hernia Repair After Prostatectomy: How to Navigate Safely. Surgical Laparoscopy, Endoscopy and Percutaneous Techniques 32(1):66-72 | Robotic Inguinal Hernia Repair |
| 353 | A systematic review of rcts evaluating laparoscopic repair of inguinal hernias with self-gripping mesh versus mechanically fixed 'lightweight' mesh in the treatment of medium to large sized defects. Surgical Endoscopy and Other Interventional Techniques 32(1):S139 | review |
| 354 | A systematic review of randomised control trials assessing mesh fixation in open inguinal hernia repair. Hernia 18(2):165-176 | review |
| 355 | Preliminary experience using fibrin glue for mesh fixation in 250 patients undergoing minilaparoscopic transabdominal preperitoneal hernia repair. J Laparoendosc Adv Surg Tech A 17(1):12-15 | Not about nonfixation of mesh |
| 356 | Repeat transabdominal preperitoneal (TAPP) mesh repair after previous anterior and posterior repair for re-recurrent inguinal hernia. Surgical Endoscopy 33:S367 | Not about fixation of mesh |
| 357 | Role of selective mesh fixation in TEP repair of inguinal hernia. Hernia 20:S210 | TEP |
| 358 | Is human fibrin sealant a possible choice for the fixation of laparoscopic inguinal hernia repair? A single center experience and the analysis of the results after 326 TAPP in two years. G Chir 34(5):309-314 | Not about nonfixation of mesh |
| 359 | Insufficiency of prosthetic posterolateral overlap related to recurrence after laparoscopic transabdominal preperitoneal inguinal hernioplasty, as assessed by video review. BMC Surg 20(1):27 | Not about nonfixation of mesh |
| 360 | Autologous fibrin sealant (Vivostat ®) for mesh fixation in laparospic transabdominal preperitoneal hernia repair. Endoscopy 38(8):841-844 | Not about nonfixation of mesh |
| 361 | Autologous fibrin sealant (Vivostat) for mesh fixation in laparoscopic transabdominal preperitoneal hernia repair. Endoscopy 38(8):841-844 | Not about nonfixation of mesh |
| 362 | Bulging of the mesh after laparoscopic repair of ventral and incisional hernias. Jsls 14(4):541-546 | Not inguinal hernia |
| 363 | Chronic pain after laparoscopic transabdominal preperitoneal hernia repair: a randomized comparison of light and extralight titanized polypropylene mesh. World J Surg 35(2):302-310 | Not about nonfixation of mesh |
| 364 | Comparison of a 35g/m2 with a 16 g/m2 titanized polypropylene mesh in hernia repair in tapp technique. Surgical Endoscopy and Other Interventional Techniques 26:S8 | Not about fixation of mesh |
| 365 | Biomechanical analyses of mesh fixation in TAPP and TEP hernia repair. Surg Endosc 22(3):731-738 | Not about nonfixation of mesh |
| 366 | Laparoscopic hydrocelectomy of the canal of Nuck in adult female: Case report and literature review. Int J Surg Case Rep 66:338-341 | Case report |
| 367 | Totally extraperitoneal single incision laparoscopic inguinal hernia repair. Surgical Endoscopy and Other Interventional Techniques 26:S321 | TEP |
| 368 | NBCA (n-butyl-2-cyanoacrylate) medical adhesive for mesh fixation in inguinal herniorrhaphy (Lichtenstein, TAPP or TEP). Hernia 21(2):S143-S144 | Not about nonfixation of mesh |
| 369 | Chemical medical glue for mesh fixation in inguinal hernia repair (Lichtenstein, Tapp or Tep). Hernia 22(1):S116 | Not about nonfixation of mesh |
| 370 | Nbca (N-Butyl-2-Cyanoacrylate) medical adhesive for mesh fixation in inguinal herniorrhaphy (lichtenstein, tapp or tep). Hernia 21(1):S56 | Not about nonfixation of mesh |
| 371 | Efficacy and safety of N-butyl-2-cyanoacrylate (NBCA) medical adhesive for patch fixation in totally extraperitoneal prosthesis (TEP): a prospective, randomized, controlled trial. Hernia 20:S168‐S169 | TEP |
| 372 | Effects of intraoperative fixation of residual hernia sac on postoperative seroma in laparoscopic transabdominal preperitoneal inguinal hernia repair: a prospective randomized controlled trial. Updates in surgery | Not about nonfixation of mesh |
| 373 | Effects of intraoperative fixation of residual hernia sac on postoperative seroma in laparoscopic transabdominal preperitoneal inguinal hernia repair: a prospective randomized controlled trial. Updates Surg | Not about nonfixation of mesh |
| 374 | Fibrin glue versus staple for mesh fixation in laparoscopic transabdominal preperitoneal repair of inguinal hernia: a meta-analysis and systematic review. Surg Endosc 31(2):527-537 | review |
| 375 | Mesh fixation during laparoscopic repair of groin hernias with articulating fixation device: Short term follow-up. Hernia 22(1):S127 | Not about nonfixation of mesh |
| 376 | Light weight composite mesh for laparoscopic tep inguinal hernia repair: Air of caution. Surgical Endoscopy and Other Interventional Techniques 26:S57 | TEP |
| 377 | Feasibility and safety profile of laparoscopic inguinal hernia repair with a modular mesh. Surgical Endoscopy and Other Interventional Techniques 31(2):S66 | Not about nonfixation of mesh |
| 378 | Adhesional small bowel obstruction related to stapling device from previous laparoscopic inguinal hernia repair. BMJ Case Rep 12(4) | Case report |
| 379 | International guidelines for groin hernia management. Hernia 22(1):1-165 | guidelines |
| 380 | A novel approach for the treatment of Morgagni hernias: robotic transabdominal preperitoneal diaphragmatic hernia repair. Hernia 26(1):355-361 | robotic hernia repair |
| 381 | Laparoscopic treatment of inguinal hernia: a multicenter prospective comparison of self-adhesive mesh vs traumatic fixation: clinical and economical assessment. Hernia 22(1):S97 | Not about nonfixation of mesh |
| 382 | Clinical outcome of synthetic glue vs taks fixation of the mesh during laparoscopic repair of inguinal hernia. Surgical Endoscopy and Other Interventional Techniques 28:S185 | Not about nonfixation of mesh |
| 383 | Three-point mesh fixation in robot-assisted transabdominal preperitoneal (R-TAPP) repair of 208 inguinal hernias: preliminary results of a single-center consecutive series. Langenbecks Arch Surg 407(6):2555-2561 | robotic hernia repair |
| 384 | Sutureless laparoscopic extraperitoneal inguinal herniorrhaphy using reusable instruments: two hundred three repairs without recurrence. Surg Laparosc Endosc Percutan Tech 10(1):24-29 | Not about nonfixation of mesh |
| 385 | Short learning curve for self-adhering mesh in laparoscopic inguinal hernia repair. Surgical Endoscopy and Other Interventional Techniques 32(1):S143 | Not about nonfixation of mesh |
| 386 | Technique of mobilization and mesh fixation in laparoscopic hernia repair by transabdominal preperitoneal mesh prosthesis. Dig Surg 19(6):500-501 | No comparison |
| 387 | A prospective comparison of tacks and glue for mesh fixation in laparoscopic repair of inguinal hernia. Surgical Endoscopy and Other Interventional Techniques 26:S327 | Not about nonfixation of mesh |
| 388 | Could we reduce adhesions to the intra-abdominal mesh in the first week? Experimental study with different methods of fixation. Hernia 24(6):1245-1251 | Not about nonfixation of mesh |
| 389 | A randomized controlled trial of staple fixation versus N-butyl-2-cyanoacrylate fixation in laparoscopic inguinal hernia repair. Chotmaihet thangphaet [Journal of the Medical Association of Thailand] 96 Suppl 3:S8‐13 | Not about nonfixation of mesh |
| 390 | [Complications after laparoscopic surgery of inguinal hernias]. Rozhl Chir 82(12):628-633 | Not about fixation of mesh |
| 391 | Automatic surgical phase recognition in laparoscopic inguinal hernia repair with artificial intelligence. Hernia 26(6):1669-1678 | Not about fixation of mesh |
| 392 | Single incision laparoscopic transabdominal preperitoneal mesh hernioplasty for inguinal hernia in 290 japanese patients. Surgical Endoscopy and Other Interventional Techniques 32(1):S138 | Not about fixation of mesh |
| 393 | Single incision transabdominal preperitoneal patch plasty in 60 japanese patients with inguinal hernia. Surgical Endoscopy and Other Interventional Techniques 27:S383 | Not about fixation of mesh |
| 394 | Single incision laparoscopic transabdominal preperitoneal mesh hernioplasty for inguinal hernia in 100 japanese patients. Surgical Endoscopy and Other Interventional Techniques 28:354 | Not about fixation of mesh |
| 395 | Single incision TAPP for inguinal hernia in 150 patients. Surgical Endoscopy and Other Interventional Techniques 29:S328 | Not about fixation of mesh |
| 396 | Single incision laparoscopic transabdominal preperitoneal mesh hernioplasty for complicated inguinal hernia. Surgical Endoscopy and Other Interventional Techniques 30:S413 | Not about fixation of mesh |
| 397 | Single incision laparoscopic transabdominal preperitoneal mesh hernioplasty for inguinal hernia in 250 Japanese patients. Surgical Endoscopy and Other Interventional Techniques 31:S149 | Not about fixation of mesh |
| 398 | Feasibility and safety of laparoscopic inguinal hernia repair: Is fixation essential? British Journal of Surgery 107(SUPPL 4):84 | Retrospective study |
| 399 | Laparoscopic inguinal hernia repair without mesh fixation, early results of a large randomised clinical trial. Surgical endoscopy 22(3):757‐762 | TEP |
| 400 | Mesh-fixation technique for inguinal hernia repair: umbrella review. BJS Open 6(4) | review |
| 401 | Cost-effectiveness analysis of mesh fixation techniques for laparoscopic and open inguinal hernia surgeries. BMC Health Serv Res 22(1):1125 | Not about nonfixation of mesh |
| 402 | A meta-analysis of randomized controlled trials of fixation versus nonfixation of mesh in laparoscopic total extraperitoneal inguinal hernia repair. Surgical endoscopy 25(9):2849‐2858 | review |
| 403 | TAPP and IPOM in a patient with inguinal and incisional ventral hernia L3W2. Surgical Endoscopy and Other Interventional Techniques 31(2):S195 | Case report |
| 404 | Laparoscopic hernia repair with self-gripping mesh - A novel technique. Surgical Endoscopy and Other Interventional Techniques 25:S153 | Not about fixation of mesh |
| 405 | International guidelines for groin hernia management. Hernia:1-165 | guidelines |
| 406 | [Application of Da Vinci robotic surgery to hernia repair]. Zhonghua Wei Chang Wai Ke Za Zhi 21(7):740-743 | robotic surgery |
| 407 | Early clinical outcomes following laparoscopic inguinal hernia repair. Dan Med J 60(7):B4672 | Not about fixation of mesh |
| 408 | Randomized clinical trial of fibrin glue versus tacked fixation in laparoscopic groin hernia repair. Surg Endosc 27(8):2727-2733 | Not about nonfixation of mesh |
| 409 | Tisseel vs tack staples as mesh fixation in totally extraperitoneal laparoscopic repair of groin hernias: A retrospective analysis. Surgical Endoscopy and Other Interventional Techniques 19(5):724-727 | TEP |
| 410 | Tisseel versus tack staples as mesh fixation in totally extraperitoneal laparoscopic repair of groin hernias: a retrospective analysis. Surg Endosc 19(5):724-727 | TEP |
| T411 | Comparison of postoperative pain in laparoscopic inguinal hernia repairs by the transabdominal preperitoneal technique with self-gripping mesh versus tacker fixation. International journal of abdominal wall and hernia surgery 3(2):45‐49 | Not about nonfixation of mesh |
| 412 | Comparison of postoperative pain in laparoscopic inguinal hernia repairs by the transabdominal preperitoneal technique with self-gripping mesh versus tacker fixation. International Journal of Abdominal Wall and Hernia Surgery 3(2):45-49 | Not about nonfixation of mesh |
| 413 | Evaluation of the Rebound Hernia Repair device for laparoscopic hernia repair. Jsls 14(1):95-102 | Not about nonfixation of mesh |
| 414 | AHRQ Comparative Effectiveness Reviews. Surgical Options for Inguinal Hernia: Comparative Effectiveness Review. Agency for Healthcare Research and Quality (US), Rockville (MD) | review |
| 415 | Novel technique of mesh fixation with cyanoacrylate in totally extraperitoneal laparoscopic hernia repair: early experience. J Med Assoc Thai 95 Suppl 3:S98-101 | TEP |
| 416 | LAPAROSCOPIC INGUINAL HERNIOPLASTY USING TRANSABDOMINAL PREPERITONEAL APPROACH (TAPP) IN YOUNG SURGEONS: A PERSONAL EXPERIENCE. British Journal of Surgery 109:vii28 | Not about fixation of mesh |
| 417 | Novel laparoscopic methods for inguinal hernia post pelvic fracture: A case report. Int J Surg Case Rep 67:173-177 | Case report |
| 418 | Suture Mesh Fixation versus Tacker Mesh Fixation in Laparoscopic Inguinal Hernia Repair. International Journal of Pharmaceutical and Clinical Research 15(3):1-6 | Not about nonfixation of mesh |
| 419 | The significance of fixation vs. non-fixation of prolene mesh placed in preperitoneal space of rats in T.E.P procedure. European Surgical Research 50:98 | TEP |
| 420 | TAPP using needlescopic instruments for inguinal hernia in adults. Surgical Endoscopy and Other Interventional Techniques 29:S79 | Not about fixation of mesh |
| 421 | Minilaparoscopic inguinal hernia repair (M-TAPP) with 5mm laparoscope and 2mm grasper: An alternative to traditional TAPP. Surgical Endoscopy and Other Interventional Techniques 30:S404 | Not about fixation of mesh |
| 422 | Micro-laparoscopic inguinal hernia repair (M-TAPP): An alternative to traditional TAPP. Surgical Endoscopy and Other Interventional Techniques 30:S66 | Not about fixation of mesh |
| 423 | Early assessment of bilateral inguinal hernia repair: a comparison between the laparoscopic total extraperitoneal and Stoppa approaches. Journal of minimal access surgery 12(3):271‐277 | TEP |
| 424 | Laparoscopic transperitoneal versus extraperitoneal inguinal hernia repair: a prospective clinical trial. Acta Chir Belg 98(3):132-135 | Not about fixation of mesh |
| 425 | Robotic inguinal hernia repair eliminates the need for post-operative narcotics and demonstrates lower post-operative pain scores. Hernia 22(1):S183 | Robotic inguinal hernia repair |
| 426 | 3D technology as a preoperative assesment on patients with hernias of the groin. A quantitative classification proposal aimed to reach surgical approaching. European Journal of Surgical Oncology 48(2):e146 | Not about fixation of mesh |
| 427 | Early outcomes of Adhesix® mesh compared to a variety of mechanically secured meshes for abdominal and inguinal hernias. Surgical Endoscopy and Other Interventional Techniques 30:S405 | Not about fixation of mesh |
| 428 | Totally preperitoneal laparoscopic inguinal herniorrhaphy using balloon distention. Scand J Gastroenterol Suppl 208:67-73 | TEP |
| 429 | Totally preperitoneal laparoscopic inguinal herniorrhaphy using balloon distention. Scandinavian Journal of Gastroenterology, Supplement 30(208):67-73 | TEP |
| 430 | Laparoscopic transabdominal preperitoneal inguinal hernia repair using needlescopic instruments: a 15-year, single-center experience in 317 patients. Surg Endosc 26(7):1898-1902 | Not about fixation of mesh |
| 431 | Single incision totally extra peritoneal inguinal hernia repair with self-fixating mesh. Surgical Endoscopy and Other Interventional Techniques 27:S58 | TEP |
| 432 | A novel technique of single incision endoscopic totally extraperitoneal hernia surgery under local anesthesia. Surgical Endoscopy and Other Interventional Techniques 29:S86 | TEP |
| 433 | Topic: Inguinal Hernia - Fixation. Hernia 19 Suppl 1:S254-260 | No comparison |
| 434 | A prospective study on effects of treatment of hernia sac stump in laparoscopic transabdominal preperitoneal inguinal hernia repair on postoperative seroma. Chinese Journal of Digestive Surgery 19(1):81-86 | Not about fixation of mesh |
| 435 | Different types of mesh fixation for laparoscopic repair of inguinal hernia: A protocol for systematic review and network meta-analysis with randomized controlled trials. Medicine (Baltimore) 97(16):e0423 | Review |
| 436 | [HerniaSurge: international guidelines on treatment of inguinal hernia in adults : Comments of the Surgical Working Group Hernia (CAH/DGAV) and the German Hernia Society (DHG) on the most important recommendations]. Chirurg 89(8):631-638 | Guidelines |
| 437 | Early experience with a new anatomical mesh for endo-laparoscopic inguinal hernia repair. Surgical Endoscopy and Other Interventional Techniques 29:S76 | Not about fixation of mesh |
| 438 | Endo-laparoscopic inguinal hernia repair in female patients: Benefits and results. Surgical Endoscopy and Other Interventional Techniques 30:S412 | Not about fixation of mesh |
| 439 | Titanium Vs. absorbable spiral tackers fixation in totally extraperitoneal (tep) inguinal hernia repair. Surgical Endoscopy and Other Interventional Techniques 31(2):S314 | TEP |
| 440 | Long-term outcome of a new 3D anatomical mesh for endo-laparoscopic inguinal hernia repair. Surgical Endoscopy and Other Interventional Techniques 30:S67 | Not about fixation of mesh |
| 441 | Early experience with a new anatomical mesh for endo-laparoscopic inguinal hernia repair. Surgical Endoscopy and Other Interventional Techniques 28:353 | Not about fixation of mesh |
| 442 | Utility of magnetic resonance imaging to monitor meshes: Correlating imaging and clinical outcome of patients undergoing inguinal hernia repair. Experience from more than 70 MRI. Hernia 20:S169-S170 | Not about fixation of mesh |
| 443 | Laparoscopic transabdominal preperitoneal (TAPP) groin hernia repair using n-butyl-2-cyanoacrylate (Liquiband®Fix8™) for mesh fixation and peritoneal closure: learning experience during introduction into clinical practice. Hernia 23(3):601-613 | Not about fixation of mesh |
| 444 | Long-term outcome and chronic pain in atraumatic fibrin glue versus staple fixation of extra light titanized meshes in laparoscopic inguinal hernia repair (TAPP): a single-center experience. Surg Endosc 34(5):1929-1938 | Not about nonfixation of mesh |
| 445 | Femoral nerve injury following transabdominal preperitoneal inguinal hernia repair: A case report. International Journal of Abdominal Wall and Hernia Surgery 5(4):204-208 | Case report |
| 446 | Two cases of early recurrence after transabdominal preperitoneal inguinal hernia repair. Asian J Endosc Surg 11(1):71-74 | Case report |
| 447 | Laparoscopic repair of inguinal hernia in adults. Ann Transl Med 4(20):402 | Review |
| 448 | [Modified laparoscopic transabdominal preperitoneal repair of groin hernias]. Nan Fang Yi Ke Da Xue Xue Bao 28(12):2272-2273 | No comparison |
| 449 | IMPLEMENTATION OF LAPAROSCOPIC INGUINAL HERNIA REPAIR IN A LOCAL HOSPITAL. 2 YEARS' EXPERIENCE AND CHALLENGES. British Journal of Surgery 110:ii29 | No comparison |
| 450 | The effect of mesh fixation on migration and postoperative pain in laparoscopic TEP repair: prospective randomized double-blinded controlled study. Hernia 27(1):63‐70 | TEP |
| 451 | MESH FIXATION IN LAPAROENDSOCOPIC REPAIR OF LARGE M3 INGUINAL HERNIAS - MULTICENTER, DOUBLE-BLINDED, RANDOMIZED TRIAL-> MEFI TRIAL. British Journal of Surgery 110:ii25 | Conference Abstract |
| 452 | Is mesh fixation necessary in laparoendoscopic techniques for M3 inguinal defects? An experimental study. Surg Endosc 37(3):1781-1788 | experimental study  no perioperative outcome |
| 453 | Multicentric evaluation by Verbal Rate Scale and EuroQoL-5D of early and late post-operative pain after TAPP and TEP procedures with mechanical fixation for bilateral inguinal hernias. Ann Ital Chir 82(6):437-442 | Not about nonfixation |
| 454 | Prospective study: Is mesh fixation necessary for laparoscopic repair of recurrent inguinal hernias? Surgical Endoscopy and Other Interventional Techniques 26:S130 | No comparison |
| 455 | Laparoscopic management of an inguinal fecal fistula after bilateral TAPP repair. Surgical Endoscopy and Other Interventional Techniques 29:S267 | Not about fixation of mesh |
| 456 | TAPP inguinal hernia repair without mesh fixation technique for recurrent hernias: A prospective analysis. Surgical Endoscopy and Other Interventional Techniques 25:S322 | No comparison |
| 457 | Prospective evaluation of laparoscopic inguinal hernia repair without mesh fixation technique. Surgical Endoscopy and Other Interventional Techniques 25:S61 | No comparison |
| 458 | Prospective evaluation of laparoscopic inguinal hernia repair without mesh fixation technique. Surgical Endoscopy and Other Interventional Techniques 24(1):S617-S618 | No comparison |
| 459 | Totally extraperitoneal laparoscopic hernioplasty: the optimal surgical approach. Surg Laparosc Endosc Percutan Tech 19(6):501-505 | TEP |
| 460 | Laparoscopic inguinal hernia repair using an anatomically contoured three-dimensional mesh without fixation: An analysis of 47 cases. Journal of Dalian Medical University 35(1):46-49 | Not about fixation of mesh |
| 461 | A study of the “Swiss-roll” folding method for placement of self-gripping mesh in TAPP. Minimally Invasive Therapy and Allied Technologies:1-7 | Not about fixation of mesh |
| 462 | A study of the "Swiss-roll" folding method for placement of self-gripping mesh in TAPP. Minim Invasive Ther Allied Technol 31(2):262-268 | Not about fixation of mesh |
| 463 | Use of human fibrin glue (tissucol) versus staples for mesh fixation in laparoscopic transabdominal preperitoneal hernioplasty [2]. Annals of Surgery 246(5):903-904 | Not about nonfixation of mesh |
