# Supplementary material for: Non-fixation versus fixation of mesh in laparoscopic transabdominal preperitoneal repair of inguinal hernia: A systematic review and meta-analysis of randomized controlled trials
Source: PLoS One. 2024 Dec 6;19(12):e0314334. doi: 10.1371/journal.pone.0314334 (PMC11623461; doi:10.1371/journal.pone.0314334)
Supplement: S2 File — (DOCX) [file pone.0314334.s004.docx]

| **Domain of bias** | **Kalidarei[1]** | **Li[4]** | **Meshkati[5]** |
| --- | --- | --- | --- |
| bias arising from the randomization process | 1 | 0 | 0 |
| bias due to deviations from intended interventions | 0 | 0 | 0 |
| bias due to missing outcome data | 0 | 0 | 0 |
| bias in measurement of the outcome | 0 | 0 | 1 |
| bias in selection of the reported result | 0 | 0 | 0 |
| Overall risk of bias | 1 | 0 | 1 |

**Table 1. Risk of bias of included studies for postoperative pain at 6 months**

0 = low risk, 1 = some concerns, 2 = high risk

**Table 2. Risk of bias of included studies for wound and mesh infection**

| **Domain of bias** | **Habeeb[6]** | **Kalidarei[1]** | **Li[4]** | **Smith[2]** |
| --- | --- | --- | --- | --- |
| bias arising from the randomization process | 1 | 1 | 0 | 1 |
| bias due to deviations from intended interventions | 0 | 0 | 0 | 0 |
| bias due to missing outcome data | 0 | 0 | 0 | 1 |
| bias in measurement of the outcome | 0 | 0 | 0 | 0 |
| bias in selection of the reported result | 0 | 0 | 0 | 0 |
| Overall risk of bias | 1 | 1 | 0 | 2 |

0 = low risk, 1 = some concerns, 2 = high risk

**Table 3. Risk of bias of included studies for seroma formation**

0 = low risk, 1 = some concerns, 2 = high risk

| **Domain of bias** | **Azevedo[3]** | **Habeeb[6]** | **Kalidarei[1]** | **Li[4]** | **Smith[2]** |
| --- | --- | --- | --- | --- | --- |
| bias arising from the randomization process | 0 | 1 | 1 | 0 | 1 |
| bias due to deviations from intended interventions | 0 | 0 | 0 | 0 | 0 |
| bias due to missing outcome data | 0 | 0 | 0 | 0 | 1 |
| bias in measurement of the outcome | 0 | 0 | 0 | 0 | 0 |
| bias in selection of the reported result | 0 | 0 | 0 | 0 | 0 |
| Overall risk of bias | 0 | 1 | 1 | 0 | 2 |

**Table 4. Risk of bias of included studies for time to normal activity**

| **Domain of bias** | **Kalidarei[1]** | **Meshkati[5]** | **Smith[2]** |
| --- | --- | --- | --- |
| bias arising from the randomization process | 1 | 0 | 1 |
| bias due to deviations from intended interventions | 0 | 0 | 0 |
| bias due to missing outcome data | 0 | 0 | 1 |
| bias in measurement of the outcome | 0 | 1 | 1 |
| bias in selection of the reported result | 0 | 0 | 0 |
| Overall risk of bias | 1 | 1 | 2 |

0 = low risk, 1 = some concerns, 2 = high risk

**[1]**Kalidarei B, Mahmoodieh M, Sharbu Z. Comparison of mesh fixation and nonfixation in laparoscopic transabdominal preperitoneal repair of inguinal hernia. Formosan J Surgery. 2019;52: 212-220.

**[2]**Smith AI, Royston CM, Sedman PC. Stapled and nonstapled laparoscopic transabdominal preperitoneal (TAPP) inguinal hernia repair. A prospective randomized trial. Surg Endosc. 1999;13: 804-806.

**[3]**Azevedo MA, Oliveira GBT, Malheiros CA, Roll S. Are there differences in chronic pain after laparoscopic inguinal hernia repair using the transabdominal technique comparing with fixation of the mesh with staples, with glue or without fixation? A clinal randomized, double-blind trial. Arq Bras Cir Dig. 2022;35: e1670.

**[4]**Li W, Sun D, Sun Y, Cen Y, Li S, Xu Q, et al. The effect of transabdominal preperitoneal (TAPP) inguinal hernioplasty on chronic pain and quality of life of patients: mesh fixation versus non-fixation. Surg Endosc. 2017;31: 4238-4243.

**[5]**Meshkati Yazd SM, Kiany F, Shahriarirad R, Kamran H, Karoobi M, Mehri G. Comparison of mesh fixation and non-fixation in transabdominal preperitoneal (TAPP) inguinal hernia repair: a randomized control trial. Surg Endosc. 2023;37: 5847-5854.

**[6]**Habeeb T, Mokhtar MM, Sieda B, Osman G, Ibrahim A, Metwalli AM, et al. Changing the innate consensus about mesh fixation in trans-abdominal preperitoneal laparoscopic inguinal hernioplasty in adults: Short and long term outcome. Randomized controlled clinical trial. Int J Surg. 2020;83: 117-124.
