# Supplementary material for: Non-fixation versus fixation of mesh in laparoscopic transabdominal preperitoneal repair of inguinal hernia: A systematic review and meta-analysis of randomized controlled trials
Source: PLoS One. 2024 Dec 6;19(12):e0314334. doi: 10.1371/journal.pone.0314334 (PMC11623461; doi:10.1371/journal.pone.0314334)
Supplement: S1 Data — (DOCX) [file pone.0314334.s005.docx]

**Table 1 Recurrence data for meta-analysis**

| Trial | Recurrence | |
| --- | --- | --- |
|  | Non-fixation group (n) | Fixation group (n) |
| Smith et al | 0 (263) | 3 (273) |
| Li et al | 0 (50) | 0 (50) |
| Kalidarei et al | 2 (48) | 0 (48) |
| Habeeb et al | 2 (266) | 3 (532) |
| Azevedo et al | 0 (21) | 0 (42) |
| Meshkati et al | 0 (50) | 1 (50) |

**Table 2 Postoperative pain at 6 months**

| Trial | Postoperative pain at 6 months (mean VAS±SD) | |
| --- | --- | --- |
|  | Non-fixation group (n) | Fixation group (n) |
| Li et al | 0.8±0.3 (50) | 1.0±0.1 (50) |
| Kalidarei et al | 0.26±0.59 (39) | 0.66±1.17 (41) |
| Meshkati et al | 0.02±0.14 (50)^a^ | 0.12±0.33 (50)^a^ |

a Original data were provided as median and interquartile range, mean and standard deviation were calculated by methods described by Hozo et al^[1]^.

[1] Hozo SP, Djulbegovic B, Hozo I. Estimating the mean and variance from the median, range, and the size of a sample. BMC Med Res Methodol. 2005;5: 13.

**Table 3 Infection data for meta-analysis**

| Trial | Infection | |
| --- | --- | --- |
|  | Non-fixation group (n) | Fixation group (n) |
| Smith et al | 4 (253) | 5 (249) |
| Li et al | 0 (50) | 0 (50) |
| Kalidarei et al | 2 (39) | 0 (41) |
| Habeeb et al | 0 (266) | 0 (532) |

**Table 4 Seroma formation**

| Trial | Seroma formation | |
| --- | --- | --- |
|  | Non-fixation group (n) | Fixation group (n) |
| Smith et al | 35 (234) | 24 (213) |
| Li et al | 4 (50) | 5 (50) |
| Kalidarei et al | 2 (39) | 3 (41) |
| Habeeb et al | 1 (266) | 17 (532) |
| Azevedo et al | 2 (21) | 3 (42) |

**Table 5 Time to normal activity**

| Trial | Time to normal activity (mean±SD) | |
| --- | --- | --- |
|  | Non-fixation group (n) | Fixation group (n) |
| Smith et al | 12±9.17 (253) ^a^ | 13±10.83 (249) ^a^ |
| Kalidarei et al | 6.90±1.92 (39) | 8.79±2.27 (41) |
| Meshkati et al | 6±2.62 (50) ^b^ | 18±5.24 (50) ^b^ |

a Original data were provided as mean, median and range, standard deviation was calculated by methods described by Hozo et al^[1]^.

b Original data were provided as median and interquartile range, mean and standard deviation were calculated by methods described by Hozo et al^[1]^.

[1] Hozo SP, Djulbegovic B, Hozo I. Estimating the mean and variance from the median, range, and the size of a sample. BMC Med Res Methodol. 2005;5: 13.

Sensitivity analysis

**Table 6 Recurrence**

|  | RR | 95% CI | I2 |
| --- | --- | --- | --- |
| Smith’s trial excluded | 1.42 | 0.40-4.98 | 0% |
| Li’s trial excluded | 0.83 | 0.29-2.39 | 8% |
| Kalidarei’s trial excluded | 0.53 | 0.15-1.85 | 0% |
| Habeeb’s trial excluded | 0.65 | 0.17-2.46 | 31% |
| Azevedo’s trial excluded | 0.83 | 0.29-2.39 | 8% |
| Meshkati’s trial excluded | 0.96 | 0.31-2.98 | 29% |

**Table 7 Infection**

|  | RR | 95% CI | I2 |
| --- | --- | --- | --- |
| Smith’s trial excluded | 5.25 | 0.26-106.01 |  |
| Li’s trial excluded | 1.18 | 0.39-3.62 | 24% |
| Kalidarei’s trial excluded | 0.79 | 0.21-2.90 |  |
| Habeeb’s trial excluded | 1.18 | 0.39-3.62 | 24% |

**Table 8 Seroma formation**

|  | RR | 95% CI | I2 |
| --- | --- | --- | --- |
| Smith’s trial excluded | 0.47 | 0.22-1.02 | 27% |
| Li’s trial excluded | 0.95 | 0.62-1.45 | 52% |
| Kalidarei’s trial excluded | 0.95 | 0.63-1.44 | 51% |
| Habeeb’s trial excluded | 1.20 | 0.79-1.83 | 0% |
| Azevedo’s trial excluded | 0.92 | 0.61-1.39 | 53% |
