# Supplementary material for: Non-fixation versus fixation of mesh in laparoscopic transabdominal preperitoneal repair of inguinal hernia: A systematic review and meta-analysis of randomized controlled trials
Source: PLoS One. 2024 Dec 6;19(12):e0314334. doi: 10.1371/journal.pone.0314334 (PMC11623461; doi:10.1371/journal.pone.0314334)
Supplement: Attachment — Submitted filename: Response to Reviewers.docx [file pone.0314334.s006.docx]

**Lovenish** **Bains**

**Academic Editor**

**PLOS ONE**

**August 17, 2024**

Dear Bains,

Thank you for your giving us an opportunity to revise our manuscript! Now we are submitting the revised manuscript entitled “Non-fixation versus fixation of mesh in laparoscopic transabdominal preperitoneal repair of inguinal hernia: a systematic review and meta-analysis of randomized controlled trials” for consideration for publication in ***PLOS ONE***.

Your comments and those of the reviewers were helpful and enabled us to improve the quality of our manuscript. In the following pages are our point-by-point responses to each of the comments of the reviewers as well as your own comments. Revisions in manuscript are shown using yellow highlight for replacement and additions, and strikethrough font for deletions.

All authors have read and approved the re-submission of the manuscript!

Thank you for your consideration of our paper and we are looking forward to hearing from you!

Sincerely yours,

JianPing Bai, M.D

Department of General Surgery

The 983rd Hospital of Joint Logistic Support Force of PLA

60 Huangwei Road

Tianjin, China, 300142

Rebuttal Letter

**Response to reviewers:**

**Reviewer 1：**

**Reviewer point #1:** References need to be modified as per journal criteria.

**Author response #1:** Thanks for your instruction. We have checked and modified the format of reference to meet journal standards.

**Reviewer 2：**

**Reviewer point #1:** The paper is lucidly written and I enjoyed reading it, except that some subheadings are starting with small letters. Also, few reference may need formatting according to the specifications.

**Author response #1:** Thank you for your comment. And we apologize for our carelessness. These errors have been modified in the revised manuscript (Line 227, Line 253, Line 260, Line 263). The references had been checked and modified.

**Reviewer 3：**

**Reviewer point #1:** Fifteen cases were converted from Laproscopy to open. How were the statistical calculations done, in view of this observation?

**Author response #1:** Thank you for your careful review. We checked the original study. The fifteen cases which were converted from Laproscopy to open were all included in statistical calculations for outcomes such as recurrence, seroma formation.

**Reviewer point #2:** Were the scoring for pain evaluation identical for all studies?

**Author response #2:** Thank you for your careful review. The scoring for pain evaluation was identical in five included studies. We have added the detailed description of pain evaluation in outcomes section (Line 213-216).

**Reviewer point #3:** Individual Post operative complication description in detail would be more beneficial.

**Author response #3:** Thank you for your instructive suggestion. We have added individual postoperative complication descriptions in Table 2.

**Reviewer 4：**

**Reviewer point #1:** It could be useful to add in table 1 the number of patients for each study.

**Author response #1:** Thank you for your instructive suggestion. We have added the patient number of each study in Table 1.

**Reviewer point #2:** The third included study, Kalidarei B et al, has patients with recurrent inguinal hernia, please could the Authors briefly specify whether after surgery with (open, TEP or TAPP) or without mesh. Moreover, were these patients excluded from recurrence rate analysis?

**Author response #2:** Thank you for your careful review. We checked the original study. We had made a mistake that the study of Kalidarei et al. [1] did not include patients of recurrent inguinal hernia. Their criteria for inclusion were “patients within the age range of 18–50 years, those with inguinal hernia, those with no history of inguinal hernial repair surgery, and those with laparotomy and strangulated or incarcerated hernia participated in the study”. We are very sorry for this careless mistake. We have made modification in the revised manuscript (Line 163-164, Table 1).

[1] Kalidarei B, Mahmoodieh M, Sharbu Z. Comparison of mesh fixation and nonfixation in laparoscopic transabdominal preperitoneal repair of inguinal hernia. Formosan Journal of Surgery 2019;52: 212-220.

**Reviewer point #3:** Although only two studies detailed the size of hernia defect, as properly reported in the discussion (line 368-370), the same clarification should be used in the conclusion when the Authors assert that non-fixation “does not increase recurrence rate”.

**Author response #3:** Thank you for your instruction. We have modified the conclusion to make it more accurate (Line 64-65, Line 379-381).

**Reviewer 5:**

**Reviewer point #1:** Your paper is interesting and well written. Unfortunately, as you highlighted, data from Literature are heterogeneous and for several aspects is difficult to draw a conclusion. Your efforts are appreciable and the results, although partial, are interesting.

**Author response #1:** Thank you for your comment. The heterogeneity of the data from the included studies did cause difficulties in synthetic analysis. We tried our best to make accurate conclusions, which may be interesting for surgeons in this field.

**Academic Editor:**

**Reviewer point #1:** Statistical methods need clarification regarding cases converted to open surgery, and consistency in pain evaluation scoring across studies.

**Author response #1:** Thank you for your careful instruction. We reviewed the study conducted by Habeeb et al. [2] carefully. They provided the causes of converting to open surgery (adhesion in the pelvis or endometriosis in the pelvis). However, they did not provide any other details of these cases, such as which study group they came from. The fifteen cases which were converted from Laproscopy to open were all included in statistical calculations for outcomes of that study. We have modified the description of pain evaluation scoring across studies in outcomes section (Line 213-216) to clarify the consistency. Four studies with available pain score data all used the same Visual Analog Scale (VAS) pain scale.

[2] Habeeb T, Mokhtar MM, Sieda B, Osman G, Ibrahim A, Metwalli AM, et al. Changing the innate consensus about mesh fixation in trans-abdominal preperitoneal laparoscopic inguinal hernioplasty in adults: Short and long term outcome. Randomized controlled clinical trial. Int J Surg 2020;83: 117-124.

**Reviewer point #2:** Detailed postoperative complication descriptions and inclusion of patient numbers per study in Table 1 would enhance clarity.

**Author response #2:** Thank you for your instructive suggestion. We have added the patient numbers for each study in Table 1. Considering the gist of each table, we have added postoperative complication descriptions in Table 2.

**Reviewer point #3:** Addressing hernia defect size in both discussion and conclusion sections is crucial.

**Author response #3:** Thank you for your instruction. Hernia defect size is an important parameter for evaluating inguinal hernia. It is always taken into account in the treatment of hernias. Unfortunately, only two of the studies in our meta-analysis reported hernia defect size. This is indeed a limitation of our study. We have modified the conclusion to make it more accurate (Line 64-65, Line 379-381).

**Other changes:**

1. Line 46-48 of original manuscript, the statement of“The purpose of this meta-analysis is to comprehensively compare the clinical outcomes between non-fixation and fixation of mesh in TAPP to determine whether non-fixation could affect the outcomes” was modified as “The aim of this meta-analysis was to comprehensively compare the clinical outcomes of non-fixation and fixation of mesh in TAPP to determine whether non-fixation could affect the outcomes” in line 46-48 of the new version.

2. Line 50 of original manuscript, the statement of “The literature search was completed at June 22, 2023” was modified as “The literature search was completed on June 22, 2023” in line 50 of the new version.

3. Line 52-53 of original manuscript, the statement of “The primary outcome measures were recurrence, evaluation of postoperative pain” was modified as “The primary outcome measures were recurrence and evaluation of postoperative pain” in line 52-53 of the new version.

4. Line 67-68 of original manuscript, the statement of “The surgical procedure for inguinal hernia repair is primarily categorized into open repair and laparoscopic mesh repair” was modified as “Surgical procedures for inguinal hernia repair are primarily classified as open repair and laparoscopic mesh repair ” in line 69-70 of the new version.

5. Line 68 of original manuscript, the statement of “Despite open mesh repair already had low recurrence rate” was modified as “Despite open mesh repair already having low recurrence rate” in line 70 of the new version.

6. Line 77-78 of original manuscript, the statement of “However, there remains some controversy regarding mesh fixation in laparoscopic mesh repair ” was modified as “However, there is still some controversy regarding mesh fixation in laparoscopic mesh repair” in line 79-80 of the new version.

7. Line 79-80 of original manuscript, the statement of “which is believed to be an important cause of hernia recurrence after laparoscopic repair” was modified as “which is believed to be an essential cause of hernia recurrence after laparoscopic repair” in line 81-82 of the new version.

8. Line 89-90 of original manuscript, the statement of “Therefore, the influence of mesh non-fixation in TAPP remains not fully concluded ” was modified as “Therefore, the influence of mesh non-fixation in TAPP remains not sufficiently concluded ” in line 92-93 of the new version.

9. Line 135-136 of original manuscript, the statement of “All data synthesis were performed using Review Manager 5.3 software ” was modified as “All data synthesis was performed using Review Manager 5.3 software (Cochrane Collaboration, Oxford, England) ” in line 138-139 of the new version.

10. Line 156-158 of original manuscript, the statement of “There were totally 1643 patients, among which 679 patients underwent TAPP surgery with non-fixation of mesh and 964 patients with fixation of mesh ” was modified as “There were a total of 1643 patients, of which 679 patients underwent TAPP surgery with non-fixation of mesh and 964 patients with fixation of mesh ” in line 160-162 of the new version.

11. Line 158-159 of original manuscript, the statement of “the other five studies were all conducted in the near decade” was modified as “the other five studies were all conducted in the last decade ” in line 162-163 of the new version.

12. Line 163-164 of original manuscript, the statement of “The range of mean follow-up time was from 6 to 24 months ” was modified as “The mean follow-up time ranged from 6 to 24 months ” in line 167-168 of the new version.

13. Line 181 of original manuscript, the statement of “No study mentioned the use of drainage ” was modified as “None of the studies mentioned the use of drainage ” in line 181 of the new version.

14. Line 346-347 of original manuscript, the statement of “It was still uncertain that whether non-fixation of mesh in laparoscopic inguinal hernia repair could improve the return to daily activities ” was modified as “It is still uncertain whether non-fixation of mesh in laparoscopic inguinal hernia repair could improve the return to daily activities ” in line 349-350 of the new version.

15. Line 358-359 of original manuscript, the statement of “The present meta-analysis did not show any difference between non-fixation and fixation in the occurrence of infection related to surgery or the formation of seroma ” was modified as “The present meta-analysis did not show any difference between non-fixation and fixation in the occurrence of surgery-related infections or the formation of seroma ” in line 361-362 of the new version.

16. Three tables of the manuscript were all modified to follow the journal standard.
